# WT-PE: Prime editing with nuclease wild-type Cas9 enables versatile large-scale genome editing

**DOI:** 10.1038/s41392-022-00936-w

**Published:** 2022-04-20

**Authors:** Rui Tao, Yanhong Wang, Yun Hu, Yaoge Jiao, Lifang Zhou, Lurong Jiang, Li Li, Xingyu He, Min Li, Yamei Yu, Qiang Chen, Shaohua Yao

**Affiliations:** From laboratory of Biotherapy, National Key Laboratory of Biotherapy, Cancer Center, West China Hospital, Sichuan university, Renmin Nanlu 17, Chengdu, 610041 Sichuan China

**Keywords:** Gene therapy, Genetic engineering

## Abstract

Large scale genomic aberrations including duplication, deletion, translocation, and other structural changes are the cause of a subtype of hereditary genetic disorders and contribute to onset or progress of cancer. The current prime editor, PE2, consisting of Cas9-nickase and reverse transcriptase enables efficient editing of genomic deletion and insertion, however, at small scale. Here, we designed a novel prime editor by fusing reverse transcriptase (RT) to nuclease wild-type Cas9 (WT-PE) to edit large genomic fragment. WT-PE system simultaneously introduced a double strand break (DSB) and a single 3′ extended flap in the target site. Coupled with paired prime editing guide RNAs (pegRNAs) that have complementary sequences in their 3′ terminus while target different genomic regions, WT-PE produced bi-directional prime editing, which enabled efficient and versatile large-scale genome editing, including large fragment deletion up to 16.8 megabase (Mb) pairs and chromosomal translocation. Therefore, our WT-PE system has great potential to model or treat diseases related to large-fragment aberrations.

## Introduction

Aberrations of large chromosomal regions, including duplication, deletion, translocation, and other structural changes, are the cause of a subtype of hereditary genetic disorders, such as thalassemia and hemophilia A.^1–3^ They are also common somatic alterations found in human cancer cells and often function as driving forces for the disease on-set or advance.^4–7^ These aberrations dramatically alter the content and structure of the involved chromosome, leading to uncontrolled gene function or large-scale dysregulated gene expression.^5,8,9^ Although the importance of large genomic aberrations in genetic diseases and cancers has been widely recognized, our knowledge about the detailed mechanisms and treatments of these disorders is hurdled in part by lacking an efficient and precise editing tool capable of manipulating the aberrations.

Genome editing technology, especially the recently developed prime editing (PE) technique has enabled targeted introduction of multiple types of edits to the genome, including deletion, insertion, and base substitution, while without requiring the generation of double strand breaks or donor templates.^10^ Current prime editors are composed of a Cas9 nickase (H840A for spCas9) and an engineered reverse transcriptase (RT) fusion protein (PE2), a prime editing guide RNA (pegRNA), and a nick single guide RNA (sgRNA). Concerted actions of these components generate an ssDNA break (SSB) in the target site that is recognized by the prime binding site (PBS) of the pegRNA to form an RNA-DNA heteroduplex. Then the 3′ end of DNA in the duplex is extended by RT according to the information encoded by the pegRNA to form a 3′ extended flap. Eventually, the flap is integrated to the genome via endogenous DNA repair or replication mechanisms. The nick sgRNA introduces a second SSB in the opposite strand to direct DNA repair to use the edited strand as a template, thereby improving the integration of the edits to the genome.^10^ Although current prime editors are efficiency in introducing small scale DNA editing in a variety of organisms,^11–14^ their efficiency in large scale DNA manipulation remains less efficient.

Here we designed a novel prime editing system by fusing RT with nuclease wild-type Cas9 (WT-PE) to perform large scale genomic manipulation. Different from the traditional prime editor (PE2), this novel system simultaneously introduced a DSB and a 3′ extended flap at the target site, which were then integrated into the genomes by endogenous mechanisms. When it was coupled with paired pegRNAs, WT-PE achieved efficient large-scale genomes editing, including large fragment deletion and chromosomal translocation. Therefore, we anticipate that our WT-PE system may contribute to the modeling or treatment of diseases related to large-fragment aberrations.

## Results

### Design and characterization of WT-PE system

**Fig. 1:**
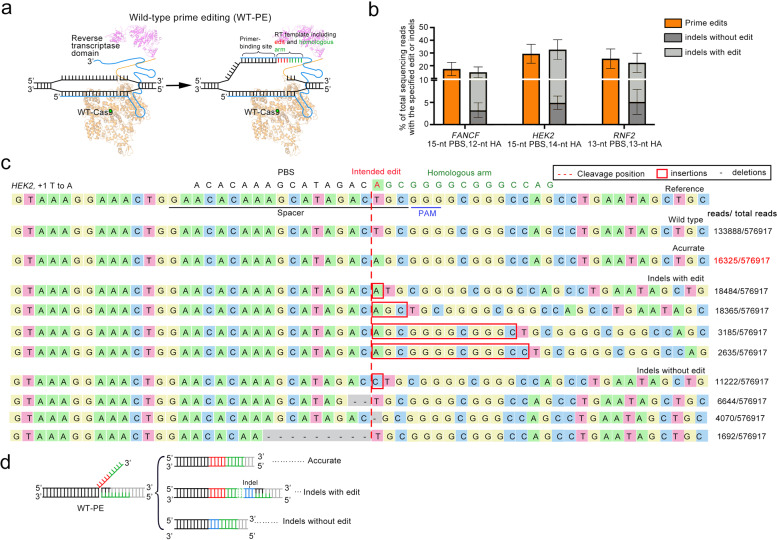
Design and characterization of WT-PE. **a** A diagram showing the putative action mode of WT-PE. Different from PE2, WT-PE uses a nuclease preserved spCas9 and introduces DSB in the target site (left panel). The broken non-target strand is then recognized by PBS of the pegRNA and then extended by RT domain in the guidance of RT template. The RT template is designed to contain aimed edit (in red) and a homologous arm (HA, in green) that is complementary to the PAM proximal end of the target strand (right panel). **b** HTS analysis of the editing outcomes of WT-PE. Three types of outcomes were observed through the HTS: accurate edits, edits containing indels and pure indels. The prime edits represented accurate edits+edits containing indels. Plots showed mean ± s.d. of three independent biological replicates. **c** Sequence alignment showing top 10 sequences of WT-PE outcomes in HEK2 locus. Desired sequence containing +1 A to T conversion served as a reference sequence with spacer and PAM sequences underlined. The position of DSB was labeled with a red dash. **d** Schematic diagram of three types of outcomes.

Previous study has demonstrated that spCas9 is a single-turnover enzyme with slow product releasement.^15^ An in vitro measurement of binding kinetics revealed that spCas9 binds to its target DNA tightly even the target has been cleaved at both strands.^16,17^ Such a binding kinetics of spCas9 intrigued us to propose a prime editing system by using nuclease wildtype spCas9 based on the hypothesis that slow product releasement of spCas9 permits sufficient time for pegRNA to hybridize with the NTS (non-target strand of sgRNA) and for RT (reverse transcriptase) to produce ssDNA extension. To test this possibility, we fused the wild-type spCas9 protein with RT to generate WT-PE expression vector (Fig. 1a) and determined its activity in HEK293T cell line by co-transfection with a panel of pegRNAs that had been verified by PE2 system to produce +5 G to T edit in *FANCF* loci, +1 T to A edit in *HEK2* loci and +1 C to A edit in *RNF2* loci. High-throughput sequencing (HTS) analysis of the target regions revealed that WT-PE did produce intended prime editing (Fig. 1b, c and Supplementary Fig. 1). However, a large portion of the edits was accompanied with unintended indels (~14.9% for *FANCF*, ~32.7% for *HEK2*, ~22.3% for *RNF2*) (Fig. 1b). These indels were likely due to the direct end joining of the extended ssDNA and PAM proximal end of DSB by nonhomologous DNA end joining (NHEJ) pathway, a prominent DSB repair pathway in high eukaryotes that tends to be error-prone,^18^ because these indels were flanked by repeated HA sequences (Fig. 1c and Supplementary Fig. 1). In addition, indels without intended edits were also observed (~3.2% for *FANCF*, ~4.9% for *HEK2*, ~5.1% for *RNF2*) in-between both ends of the DSBs, indicating that this type of indels were stemmed from the failure of prime editing to extend ssDNA (Fig. 1c, d and Supplementary Fig. 1).

The outcomes of WT-PE suggested that NHEJ occurred even in the presence of homologous region in-between the extended 3′ DNA flap and the PAM proximal DSB end, indicating that WT-PE can be re-wired to perform homology independent prime editing. To test this possibility, we removed the HA fragment from pegRNA, so that the 3′ flap was not homologous to the PAM proximal DSB end (Supplementary Fig. 2a). In addition, we added an EcoRV restriction site into the 5′ proximal portion of pegRNA RT-template to facilitate the detection of prime editing (Supplementary Fig. 2b). Co-transfection of these pegRNAs together with WT-PE plasmids did produce considerable level of prime editing across all three target sites, as evidenced by the presence of EcoRV site integration (Supplementary Fig. 2b, c).

As off-target effect is a big concern for Cas9 derived editing tools, next we sought to investigate the level of WT-PE induced off-target editing. A total of 17 off-target sites corresponding to three on-target sites (*FANCF, HEK2,* and *RNF2*) were selected for the analysis (Supplementary Table 2). As compared to nuclease wild type Cas9 (WT-Cas9), WT-PE produced comparable or slightly higher on-target editing (Supplementary Fig. 3). For the majority of detectable off-target events (9 out of 10), WT-PE produced lower editing than WT-Cas9 did. Detailed examination of off-target editing outcomes of WT-PE revealed that the majority of these edits were likely caused by DSBs without priming since no obvious 3′ flap-derived sequences were observed. Thus, these results suggested that WT-PE did not increase off-target editing as compared to WT-Cas9.

### Deletion of large genomic fragment via WT-PE

The above results established that WT-PE produced a special DSB in the target site, where the end opposite the PAM was installed with a 3′ extended ssDNA flap in the non-target strand. An in vitro study revealed that ssDNA with microhomology as short as 8-nt was enough for searching intermolecular homologous regions in the assistance of Rad51 protein.^19^ It is interesting to test whether we could utilize WT-PE produced 3′ flaps to search their homologous regions intra- or inter-molecularly so as to achieve large scale genomic manipulation.

**Fig. 2:**
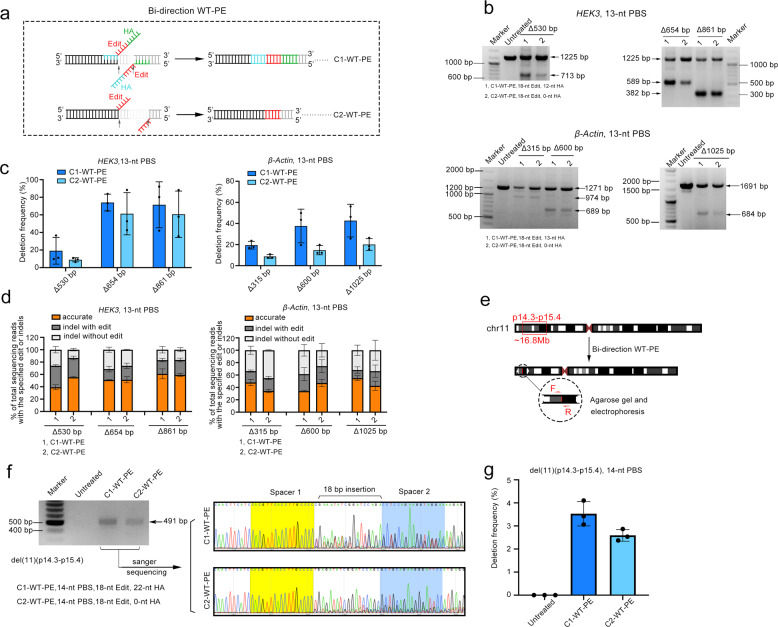
Targeted large fragment deletion by bi-directional WT-PE (bi-WT-PE). **a** A diagram showing the design of bi-directional WT-PE for targeted deletion. A pair of pegRNAs were designed to target each side sequences (black) flanking the aimed fragment to be deleted (gray). The RT template of each pegRNA in class 1 bi-directional WT-PE (C1-WT-PE) is designed to contain aimed edit (in red) and a homologous arm (HA, in green) that is complementary to the PAM proximal end of the target region of the other pegRNA (right panel). And the RT template of pegRNA in class 2 bi-directional WT-PE (C2-WT-PE) is designed to contain only the edits but complementary with each other. **b** Agarose gel analysis of the amplicons of targeted deletions. A pair of primers flanking each target deletion were used to amplify the edited region. Bands with size match wild type or edited sequences were indicated. Parameters of pegRNA, including the length of edit and HA were indicated below the gel image. **c** Quantification of the targeted deletion by photoshop software analysis of the band intensity. **d** HTS analysis of the fragments containing aimed deletions. Three types of editing outcomes were observed in WT-PE mediated deletions and their relative ratios were quantified via HTS. Plots show mean ± s.d. of three independent biological replicates. **e** Diagram showing the design of a 16.8 Mbs deletion on the short arm of chromosome 11. **f** The presence of chromosome 11 with targeted deletions was detected by PCR analysis with primers indicated in Supplementary Table 4. Left panel showed the agarose gel image of the amplicons and right panel showed their Sanger sequencing chromatograms with residue spacer sequences marked with yellow and blue. **g** Quantifying the frequencies of targeted deletions by absolute quantitative PCR. The standard curves of wildtype- or edited-chromosome-specific fragment were shown in supplementary Fig. 8. Plots showed mean ± s.d. of three independent biological replicates

As a first step to test this possibility, we designed 6 targeted intra-chromosome deletions with the length ranging from several hundreds to over one thousand base pairs. For each targeted deletion, we designed a pair of pegRNAs that directed the WT-PE to each end of the targeted deletion and induced bi-directional prime editing (Fig. 2a). The RT-template of the paired pegRNAs was made complementary to each other, harboring homologous arms (HA) (Class 1, C1) or not (Class 2, C2). As shown in Fig. 2b, we detected considerable levels of targeted deletion by both bi-directional WT-PEs across all 6 targeted deletions through PCR amplification of the sequences flanking the deletion. A quantification of the editing efficiency revealed that C1 (19.1% to 74.0%) outperformed C2 (8.7% to 61.2%) across all targets (Fig. 2c). Strikingly, the efficiency did not seem to correlate with the deletion size. To gain an insight into the detailed information of the junctions between each end, we performed HTS analysis on the fragments harboring deletion. This analysis revealed that C1-WT-PE had a comparable level of accurate editing to C2, and on average about one half of the editing events were accurate in both classes (50.4% for C1 and 55.3% for C2) (Fig. 2d). Similar to the observations on WT-PE mediated single base conversion, the rest editing events of both bi-directional WT-PEs harbored indels that either accompanied with the edits or not (Supplementary Figs. 4, 5).

After demonstrating the ability of WT-PE to induce genomic fragment deletions in HEK293T cells, we next tested its ability in other cell types. We performed 654 and 315 bp deletions at *HEK3* and *β-Actin* sites respectively in HeLa cells. The results showed that WT-PE was also efficient in deleting genomic fragments in HeLa cells. The average efficiencies of C1-WT-PE at these two sites achieved 23.1% to 8.9% respectively (Supplementary Fig. 6). Since nuclease wild type Cas9 (WT-Cas9) coupled with paired sgRNAs has been reported to induce target genomic deletion, here we compared WT-PE to WT-Cas9 by using the same paired spacers. The comparison revealed that C1-WT-PE had comparable efficiency to WT-Cas9, but the efficiency of C2-WT-PE was generally lower than those of them (Supplementary Fig. 7).

To investigate if WT-PE could perform targeted deletion at Megabase (Mb) scale, we designed a pair of pegRNAs to delete a 16.8 Mbs fragment that locates on the short arm of chromosome11 (p15.4–p14.3, Fig. 2e). PCR analysis of the sequences flanking the targeted deletion revealed that both types of WT-PE were functional (Fig. 2f). Sanger sequencing analysis of the resulting junctions identified the presence of the residue sequences of spacers and the edits in both bi-directional WT-PEs (Fig. 2f). Then we conducted absolute quantitative PCR to measure the efficiencies of targeted deletions. Two standard curves were made by using the flanking fragment of the target sites or the one containing the deletion (Supplementary Fig. 8a, b). The analysis revealed that the efficiency of C1-WT-PE (3.5%) was higher than that of C2 (2.6%) (Fig. 2g). Taken together, these results demonstrated that bi-directional WT-PE was able to perform targeted large fragment deletions up to Mb scale.

### Targeted inter-chromosomal translocation via WT-PE

Next, we sought to test the ability of WT-PE in inter-chromosome manipulating. We started the test by designing a strategy to fuse two transfected plasmid episomes, each of which harbored a verified target site of WT-PE respectively (*HEK3* and *VEGFA*) (Supplementary Fig. 9a). We co-transfected the two target plasmids with WT-PE into HEK293T cells to perform the inter-molecule manipulation. The presence of fused plasmids was detected by PCR analysis with paired primers flanking each target site located in an individual plasmid (Supplementary Fig. 9a). The analysis identified an expected fragment with size corresponding to the fusion. Sanger sequencing of the resulting fragment also identified mosaic sequences from individual target plasmids and the edits, which further confirmed the presence of fused plasmids (Supplementary Fig. 9b). Therefore, these observations established that WT-PE was able to perform inter-molecule manipulating.

**Fig. 3:**
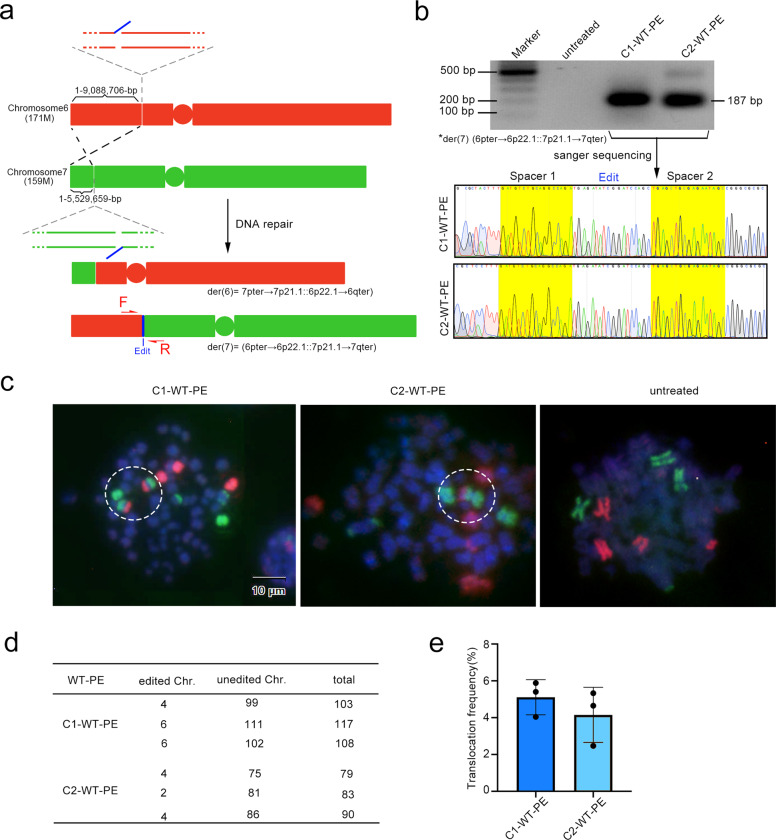
Targeted inter-chromosomal translocation by WT-PE. **a** Diagram showing the design of translocation between chromosome 6 and 7 pegRNAs were designed to cut a 171 Mbs fragment from the short arm of chromosome 6 and a 159 Mbs from the short arm of Chromosome 7. The edits that were complementary to each were installed on the short arm of chromosome 6 and the large fragment of chromosome 7 respectively, which we expected to induce translocations of (7pter→7p21.1::6p22.1→6qter) and (6pter→6p22.1::7p21.1→7qter). **b** The presence of the translocation of der (7) (6pter→6p22.1::7p21.1→7qter) was detected by PCR analysis with primers flanking each side of the translocation. Upper panel showed the agarose gel image of the resulting amplicons and lower panel showed their Sanger sequencing chromatograms with residue spacer sequences marked with yellow. However, der (6) (7pter→7p21.1::6p22.1→6qter) was not detected even with extensive PCR analysis. Primers for PCR analysis were listed in Supplementary Table 4. **c** Detecting the targeted translocation via FISH on metaphase cells. Two sets of probes covering the entire chromosome 6 (red) and 7 (green) were used for the FISH. Positive signals for der (7) (6pter→6p22.1::7p21.1→7qter) were observed highlighted in white dotted circles. No signals for der (6) (7pter→7p21.1::6p22.1→6qter) were observed. **d**, **e** Quantifications of the targeted translocations. **d** A table summarizing the translocation events that was manually counted. **e** Showed percentiles of the translocation

Inter-chromosomal translocations are frequently found and play important roles in cancers.^6^ To test the ability of WT-PE in generating inter-chromosomal translocations, we designed a strategy to exchange a portion of the short arms of chromosomes 6 and 7 (Fig. 3a). A pair of primers flanking each chromosome were designed to amplify the desired translocation. As shown in Fig. 3b, the PCR amplification identified bands with size matching the desired translocation of derivative chromosome7, der (7) (6pter→6p22.1::7p21.1→7qter), i.e., the terminal portion of the short arm (pter) of chromosome 6 to the short arm (p), band 2, sub-band 2, and sub-sub-band 1 (p22.1) attached to the short arm of chromosome 7 (p21.1) to the terminal portion of its long arm (qter), in both types of WT-PE. Surprisingly, no der (6) (7pter→7p21.1::6p22.1→6qter) was observed even upon extensive PCR analysis. Sanger sequencing of the bands also identified expected sequences of der (7) (6pter→6p22.1::7p21.1→7qter), including residue spacers of the two pegRNAs and the edits (Fig. 3b). The translocations were further demonstrated by fluorescence in situ hybridization (FISH) analysis with two sets of probes against whole chromosome 6 (red) and 7 (green). As shown in Fig. 3c, the derivative chromosome containing a portion of the short arm of chromosome 6 and the large fragment of chromosome 7 was observed in metaphase cells treated with C1 or C2 WT-PE but not in the untreated cell. Again, no derivative chromosome containing the short arm of chromosome 7 and the large fragment of chromosome 6 were observed. The frequency of hybrid chromosomes was ~5% in C1-WT-PE treated cells and ~4% in C2 treated ones (Fig. 3d, e). Similar to the result from PCR analysis, der (6) (7pter→7p21.1::6p22.1→6qter) was not observed in both types of WT-PEs. Therefore, the majority, if not all, of targeted translocations were unbalanced. The same phenomenon of unbalanced translocation was also observed in two additional chromosomal translocation events with similar strategy (Supplementary Fig. 10).

It has been demonstrated that a homologous ssDNA region longer than 8 bp is enough for intermolecular homology searching in the assistance of Rad51.^19^ We speculated that the presence of 3′ ssDNA overhang produced by WT-PE significantly improved the joining of the aimed DSB ends that located distant to each other on individual chromosomes, thereby facilitating the translocation. The failure of translocation between chromosomes without homologous ssDNAs may suggest that the efficiency of direct ligation between two distant blunt end DSBs (possibly through NHEJ) is relatively poor. Therefore, we hypothesized that coupling two sets of paired pegRNAs to WT-PE to producing matched homologous ssDNA in each break of individual chromosomes would achieve balanced translocation (Supplementary Fig. 11).

### Application of WT-PE in the correction of mutant Duchenne muscular dystrophy (DMD) gene

**Fig. 4:**
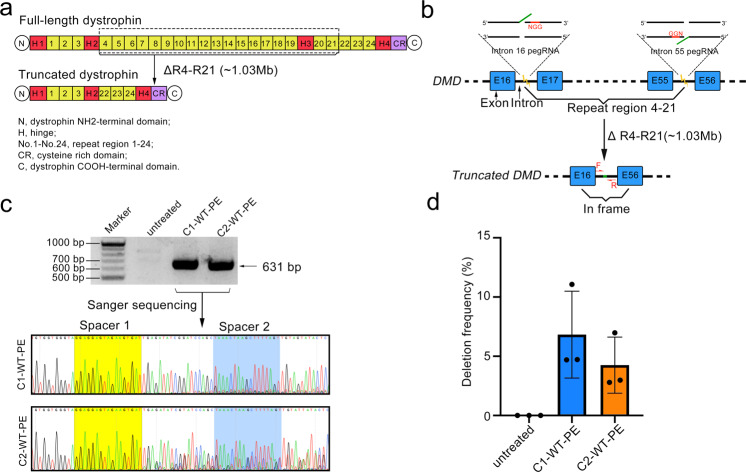
Targeted deletion of Exons 17-55 of the human *DMD* gene. **a** Diagram showing the organization of the full-length or truncated dystrophin proteins. Note that this truncated version of dystrophin has been demonstrated to be functional (ref. ^9^). Key domains of dystrophin protein were shown below the diagram. Dotted box highlighted the domains to be deleted. **b** Diagram showing the design of pegRNAs for the targeted deletion (1.03 Mbs). ssDNAs extended by WT-PE were shown in green. **c** The presence of the targeted deletion was detected by PCR analysis with primers flanking each side of the deletion shown in (**b**). Upper panel showed the agarose gel image of the resulting amplicons and lower panel showed their Sanger sequencing chromatograms with residue spacer sequences marked with yellow. Primers for PCR analysis were listed in Supplementary Table 4. **d** Quantifying the frequencies of targeted deletions by absolute quantitative PCR. The standard curves of wildtype- or edited-chromosome specific fragment were shown in supplementary Fig. 11. Plots showed mean ± s.d. of three independent biological replicates

Above observations suggested that WT-PE had the potential to treat diseases with duplications of a whole or partial gene. Besides duplication, we speculated that WT-PE can also be used to target mutations located in the redundant gene sequences but impaired the expression of that gene. Taken DMD gene as an example, this gene encoded a muscle nutrition protein, dystrophin, mutation of which leads to loss of muscle mass and motor skills.^20^ DMD is one of the largest human genes encompassing 2.6 million base pairs and has 24 functional redundant spectrin-like repeats (Fig. 4a).^21^ Mutations impairing RNA splicing or translation (pre-mature codon) within sequences encoding these redundant domains will abolish the expression of DMD.^22^ Therefore, it is possible for WT-PE to delete the mutation-containing sequences and restore DMD function. To prove this concept, we designed a pair of pegRNAs targeting intron 16 and 55 respectively to delete the sequences in-between that encode the 4th to 21st spectrin-like repeats and the 3rd hinge (Fig. 4b). Successful deletion should generate a truncated but functional dystrophin protein as previously demonstrated^23–25^ (Fig. 4a). We transfected the pegRNAs together with WT-PE into HEK293T cells and found aimed deletions by PCR analysis. As shown in Fig. 4c, primers flanking the deletion amplified fragments with expected size in both types of WT-PEs treated cells but not in untreated ones. In addition, Sanger sequencing analysis identified residue spacers of the two pegRNAs and the edits in-between, confirming the presence of targeted deletions (Fig. 4c). Quantification of the deletion frequencies with absolute quantitative PCR revealed an efficiency of ~6.8% for C1-WT-PE and ~4.3% for C2 respectively (Fig. 4d and Supplementary Fig. 12). Therefore, these results discovered a universal strategy to correct DMD mutations located in-between Exon 17 and 55, demonstrating the potential of WT-PE to treat diseases with mutations in redundant sequences or gene duplication.

## Discussion

In this work, we demonstrated that WT-PE efficiently produced prime editing, in which nuclease wild-type Cas9 instead of Cas9 nickase was used. Concerted action of wild-type Cas9 and RT introduced a double strand break (DSB) and a single 3′ extended flap in the target site, which would be integrated into the genome by endogenous repair mechanism. When WT-PE was coupled with paired prime editing guide RNAs (pegRNAs), it produced bi-directional prime editing, resulting in efficient large-scale genome editing. Recently, same prime editing strategy using nuclease wild-type Cas9 has been reported to delete large genomic fragment (up to ~10 kb).^26^ Our work showed that in addition to target deletion, WT-PE can also be rewired to produce chromosomal translocation. Together with this report, these works demonstrated that wild-type Cas9 based prime editor is a versatile editing tool in large-scale genome manipulation.

Like other genome editing tools,^27–30^ WT-PE produces designed DNA lesions that are integrated into the genome by endogenous repair mechanisms. Understanding the mechanisms responsible for the repair of intended or unintended outcomes will benefit the efficiency and specificity of WT-PE system. WT-PE system eventually leads to three types of outcomes: accurate edits, edits accompanied indels, and pure indels. From the pattern of the indels containing outcomes observed in WT-PE with HA (Fig. 1a and Supplementary Fig. 1), it is likely that NHEJ pathway partially contributes to the repair. The notion was further confirmed by editing outcomes of WT-PE without HA showing that the primed ssDNAs can be integrated into the DSBs but accompanied with indels that occurred in-between the ssDNAs and the PAM proximal end of DSBs. These observations also indicated a function of NHEJ in joining DSB ends with 3′ overhang. Although it is currently unknown which repair pathway is responsible for accurately integrating the primed ssDNAs of WT-PE or PE2, we hypothesize that undesired outcomes of WT-PE might be attenuated by inhibiting NHEJ pathway, thereby improving the rate of accurate editing.

Recently, David Liu laboratory reported that mismatch repair pathway interferes with prime editing by promoting unintended installation of insertions or deletions.^31^ They showed that attenuating mismatch repair by co-expression of siRNA against MLH1 or its dominant-negative peptide improves the fraction of intended edits, which demonstrated the notion that the outcomes of prime editing can be manipulated by modulating endogenous mechanisms.^31^ Moreover, several important progresses have also been made in enhancing the activity of prime editor itself. Equipping the prime editor with strong nuclear localization signal,^32^ modifying the 3′ engineered pegRNA to reduce self-complementing^33^ or degradation,^34^ and enhancing the RT with ssDNA binding domain^35^ were all capable of improving prime editing. It is, therefore, expectable that the introduction of these findings to WT-PE system may also increase its editing efficiency. For example, engineering 3′ extension of pegRNA is supposed to increase the portion of DSBs harboring 3′ flap in the target site, thereby reducing the unintended indels that stem from the failure of RT to install the flap. However, considering the intrinsic differences between PE2 and WT-PE in terms of the kinetics of DNA binding and editing outcomes (SSB+3′flap for PE2 and DSB+3′flap for WT-PE), these findings should be systematically examined when applying to WT-PE.

In summary, our work established that nuclease wild-type Cas9 can be used for prime editing, characterized the main features of WT-PE, and explored its versatility. Because of the special DNA lesions produced by WT-PE, DSB+3′flap, we expected its versatility beyond fragment deletions and inter-chromosomal translocations. In supplementary Fig. 13, we proposed additional utilities of WT-PE in chromosome engineering, including inner-chromosomal fragment inversion and generations of ring Chromosomes or extrachromosomal circular DNAs. Therefore, we believe that WT-PE is particularly useful for modeling or treating diseases caused by chromosomal aberrations, such as Philadelphia chromosome-related chronic myeloid leukemia and inversion-related hemophilia A.

## Materials and methods

### Plamsid construction

The plasmid WT-PE2 was constructed by one step cloning (Vazyme) with PE2 (addgene #132775). PegRNA plasmids used in this study were constructed via a two-step strategy. The first step was to construct sgRNA expression vectors (under control of human U6 promoter) by inserting spacers into Bbs1 digested sgRNA empty vector. Oligos used to generate sgRNAs were listed in (Supplementary Table 1). The second step was to construct pegRNA expression vectors via PCR-mediated end extension. Briefly, primer sets with forward primer containing U6 promoter sequences (5′ CATGTGAGGGCCTATTTCCCA 3′) and reverse primer containing the PBS+HA+polyT signal were used to amplify the sgRNA expression plasmid, and then the resulting fragment containing U6: pegRNA was cloned into the Blunt vector (YEASEN). Detailed information of pegRNAs were shown in Supplementary Table 3. All plasmids were verified by Sanger sequencing.

### Cell culture and transfection

HEK293T were cultured in Dulbecco’s modified Eagle’s medium (Thermo Fisher Scientific), supplemented with 10% (v/v) fetal bovine serum (Life Technologies) and 1% penicillin/streptomycin (Boster Biological Technology Co. Ltd.), and maintained at 37 °C with 5% CO_2_.

Cells were seeded on 96-well plate (BIOFIL) 16 h before transfection. Transfection was conducted using 390 ng of plasmids (270 ng for WT-PE, 60 ng each for two pegRNAs) and 0.7 µl of Transeasy™ (Forgene) according to the manufacturer’s protocol at a confluence of ~70–80%. Seventy-two hours post-transfection, genomic DNA was extracted by the addition of 50 µl of freshly prepared lysis buffer (10 mM Tris-HCl pH 7.5, 0.05% SDS, 25 µg ml^−1^ Proteinase K (Beyotime)). The mixture was incubated at 55 °C for 10 min and then was heat-inactivated at 95 °C for another 10 min.

### Deletion and translocation analysis

For hundreds to one thousand base pairs deletions, gDNA was subjected to PCR analysis using Phanta^®^ Max Super-Fidelity DNA Polymerase (Vazyme) and appropriate primers were listed in (Supplementary Table 4). Then the PCR amplicons were separated by agarose gel electrophoresis. Gel images were analyzed by Adobe Photoshop CC (2019) to calculate the relative greyscale of each band. Deletion efficiency was calculated as shown in Eq. 1.

1 $${{{\mathrm{Deletion}}}}\;{{{\mathrm{efficiency}}}} = \frac{{\rm{Greyscale}}\, ({\rm{deleted}}) / {\rm{base}}\, {\rm{pairs}}\, ({\rm{deleted}})}{{\rm{Greyscale}}\, ({\rm{deleted}}) / {\rm{base}}\, {\rm{pairs}}\, ({\rm{deleted}}) + {\rm{Greyscale}}\, ({\rm{undeleted}}) / {\rm{base}}\, {\rm{pairs}}\, ({\rm{undeleted}})}100{{{\mathrm{\% }}}}$$

For Megabase scale deletions and translocations, quantitative PCR was performed using the SYBR-Green (Bio-Rad) approach, qPCR primers are shown in (Supplementary Table 5). First, the flanking fragment of the target site or the one containing deletion obtained by PCR were ligated to the Blunt vector to construct reference plasmids (Supplementary Figs. 8, 10, and 11). The standard curves for the reference plasmids were determined by CT values against log-transformed concentrations of serial tenfold dilutions (2 × 10^1^, 10^2^, 10^3^, 10^4^, 10^5^, 10^6^, 10^7^, 10^8^, 10^9^ copies per 1 μL). Absolute copy numbers of flanking fragment or the one containing deletion in each gDNA were calculated with CT values based on their standard curves. Deletion efficiency was calculated as shown in Eq. 2.

2 $${{{\mathrm{Efficiency}}}} = \frac{{{{{\mathrm{copies}}}}\;{{{\mathrm{of}}}}\;{{{\mathrm{deleted}}}}\;{{{\mathrm{DNA}}}}}}{{{{{\mathrm{copies}}}}\;{{{\mathrm{of}}}}\;{{{\mathrm{flanking}}}}\;{{{\mathrm{DNA}}}}}}100{{{\mathrm{\% }}}}$$

### Targeted deep sequencing and data analysis

Genomic regions of interest were amplified by PCR with primers flanked with different barcodes (Supplementary Table 6). The products were purified with GeneJET Gel Extraction Kit (Thermo Scientific) and quantified with NanoDrop (Thermo Fisher). Samples were sequenced commercially using the Illumina Novaseq6000 platform (Personal Biotechnology, Shanghai, China). Substitution and indel frequencies were quantified as the percentage of total sequencing reads, and the threshold of editing activity was set to above 0.2%. For off-target analysis, we lowered the off-target threshold to 0.01% due to low efficiency at some sites. A custom python script provided in Supplementary Note 1 was used to analyze and quantify the efficiency of the desired edits and indels produced by WT-PE and WT-Cas9.

### Fluorescence in situ hybridization (FISH)

For preparing metaphase chromosomes, WT-PE transfected or untreated cells were treated with colcemid (final concentration 10 µg/ml) for 45 min at 37 °C. Then the cells were re-suspended and treated with hypotonic KCl solution (0.075 M, pre-warmed) at 37 °C for 10 min, washed with Ibraimov solution (5% acetic acid + 3% methanol) and fixed with fixative solution (methanol: acetic acid = 3:1). The cells were washed at least three times with fixative until it appears white. After the final wash, cells were in fixative solution. About 50 µl metaphase chromosome suspension was dropped onto a glass slide and let air dry. For chromosome painting, we used whole chromosome probes, specifically a customized XCP-mix probe (MetaSystems, Altlussheim, Germany), containing probes for chromosomes 6 and chromosomes 7. 5 μl of the mix of XCP Metasystems probe solution was applied to microscope slides (CITOGLAS) and then covered with an 18 × 18 mm^2^ coverslip (Beyotime) that was sealed with nail polish. Nuclear DNA was denatured by incubating the slides at 75 °C for 2 min, followed by overnight incubation at 37°C in a humidity chamber. Slides were washed once with 0.4 × saline sodium citrate solution (SSC; Beyotime) for 2 min at 72 °C and once with 2 × SSC supplemented with 0.05% Tween 20 (Beyotime) for 30 s at room temperature, then rinsed slide briefly in distilled water to avoid crystal formation and let air dry. DAPI (1.5 μg/mL in Anti-fluorescence quencher) was used for counterstaining.

### Statistical analyses

GraphPad Prism 8 software was used to analyze the data. All numerical values are presented as mean ± s.d. of three independent biological replicates.

## Supplementary information

Supplementary of WT-PE

## Data Availability

High-throughput sequencing data are available upon request.
