# Supplementary material for: WT-PE: Prime editing with nuclease wild-type Cas9 enables versatile large-scale genome editing
Source: Signal Transduct Target Ther. 2022 Apr 20;7:108. doi: 10.1038/s41392-022-00936-w (PMC9018734; doi:10.1038/s41392-022-00936-w)
Supplement: Supplementary file 1 — Supplementary of WT-PE [file 41392_2022_936_MOESM1_ESM.docx]

Supplementary Materials for

WT-PE：Prime editing with nuclease wild-type Cas9 enables versatile large-scale genome editing

Rui Tao^1#^, Yanhong Wang^1#^, Yun Hu^1#^, Yaoge Jiao^1^, Lifang Zhou^1^, Lurong Jiang^1^, Li Li^1^, Xingyu He^1^, Min Li^1^, Yamei Yu^1^, Qiang Chen^1^, Shaohua Yao^1^*

1. From laboratory of Biotherapy, National Key Laboratory of Biotherapy, Cancer Center, West China Hospital, Sichuan university, Renmin Nanlu 17, Chengdu 610041, Sichuan, China

# These authors contributed equally

* To whom correspondence should be addressed. Tell: 862885503628. Email: shaohuayao@scu.edu.cn

**This PDF file includes:**

Figure. S1. Sequence alignment of *HEK2* alleles edited by WT-PE (+1 T to A).

Figure. S2. Prime editing with homology independent WT-PE.

Figure. S3. Off-target analysis of WT-PE and WT-Cas9 at predicted Cas9 off-target sites.

Figure. S4. Sequence alignment of the *HEK3* alleles edited by C1-WT-PE (Δ654).

Figure. S5. Sequence alignment of the *HEK3* alleles edited by C2-WT-PE (Δ654).

Figure. S6. Deletion of large genomic fragment in Hela cells via WT-PE.

Figure. S7. Deletion efficiency of WT-PE versus WT-Cas9.

Figure. S8. Standard curves of absolute quantification of Chr11. deletion.

Figure. S9. Inter-episomal fusion by C2-WT-PE.

Figure. S10. Additional targeted inter-chromosomal translocations by WT-PE.

Figure. S11. WT-PE Strategy for balanced translocation.

Figure. S12. Standard curves of absolute quantification of *DMD* deletion.

Figure. S13. Applications of WT-PE in multiple chromosomal manipulations.

Tables S1. List of the targets tested in this study.

Tables S2. List of the off-targets tested in this study

Tables S3. Sequences of pegRNAs used for in vitro experiments.

Tables S4. Summary of primers for amplification of each target sites.

Tables S5. Primers used for qPCR to detect the copy number of target DNA fragments.

Tables S6. HTS primers used for mammalian cell genomic DNA amplification.

Supplementary Note 1. Custom python script for HTS data analysis.


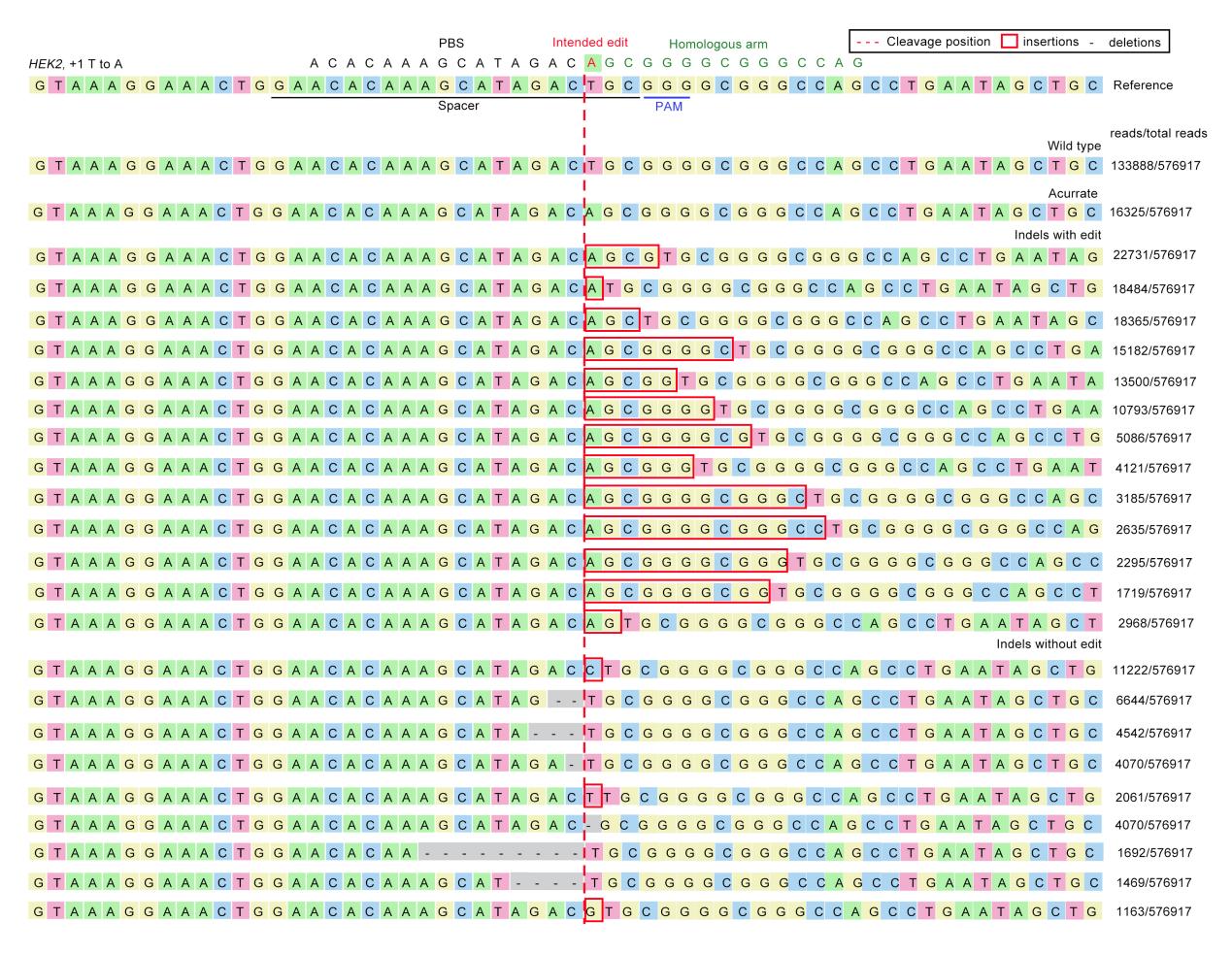


**Figure. S1. Sequence alignment of *HEK2* alleles edited by WT-PE (+1 T to A).**

Wildtype *HEK3* sequence was used as a reference with sgRNA spacer and PAM sequences underlined. HTS sequencing reads were aligned to the reference sequence and top sequences with a ratio over 0.2% were shown. Vertical dashed line marked the position of Cas9 induced DSBs. Red box marked the sequences encoded by the RT-template.

**
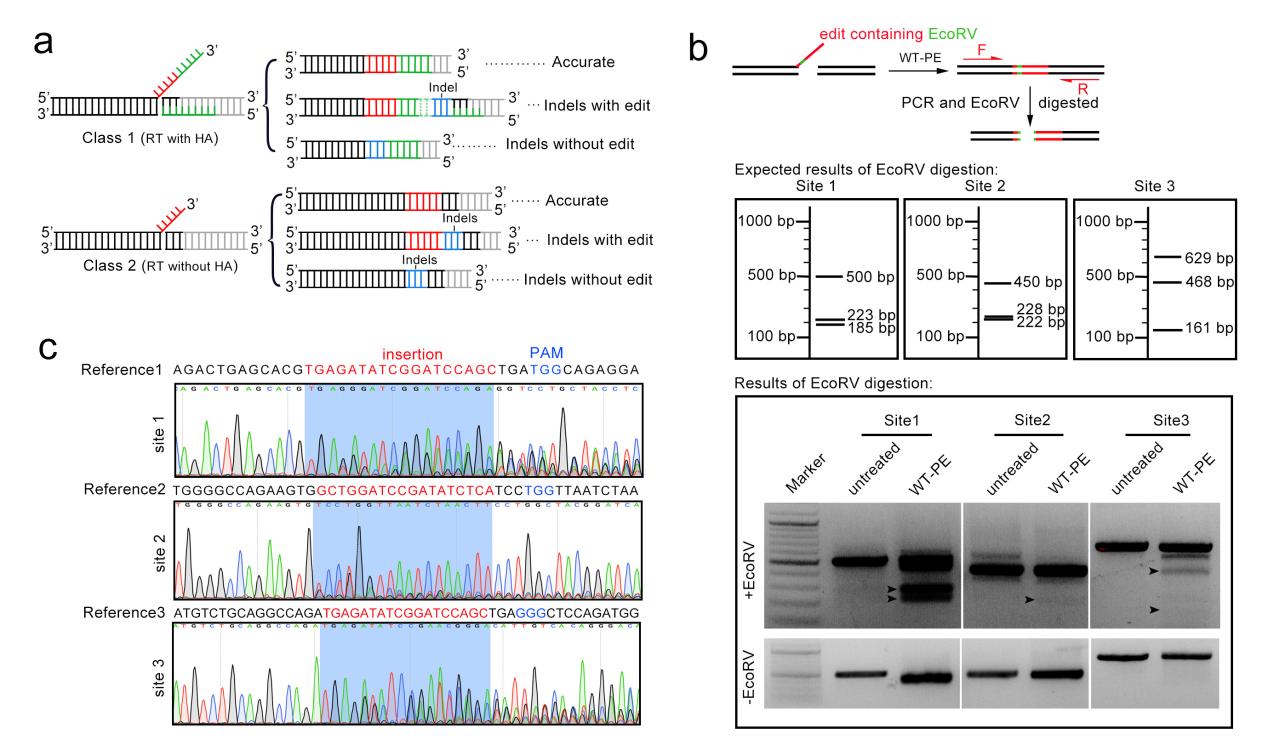
**

**Figure. S2. Prime editing with homology independent WT-PE.**

**a.** Diagram showing the putative working model of WT-PE with or without HA sequences. The edit was shown in red and HA and its complementary sequences were shown in green. **b.** Prime editing produced by homology independent WT-PE. Upper panel showed the design of the homology independent WT-PE. An EcoRV site was inserted into the RT-template so as to facilitate the detection of prime editing. Middle panel showed theoretical fragment patterns of targeted amplicons undergoing EcoRV digestion. Lower panel showed the gel image of EcoRV digested amplicons. Arrow heads pointed to the bands digested by EcoRV. Primers for PCR analysis were listed in Tables S3. **c.** Sanger sequencing chromatograph of each WT-PE edited endogenous site, with wild-type reference sequences shown on top.


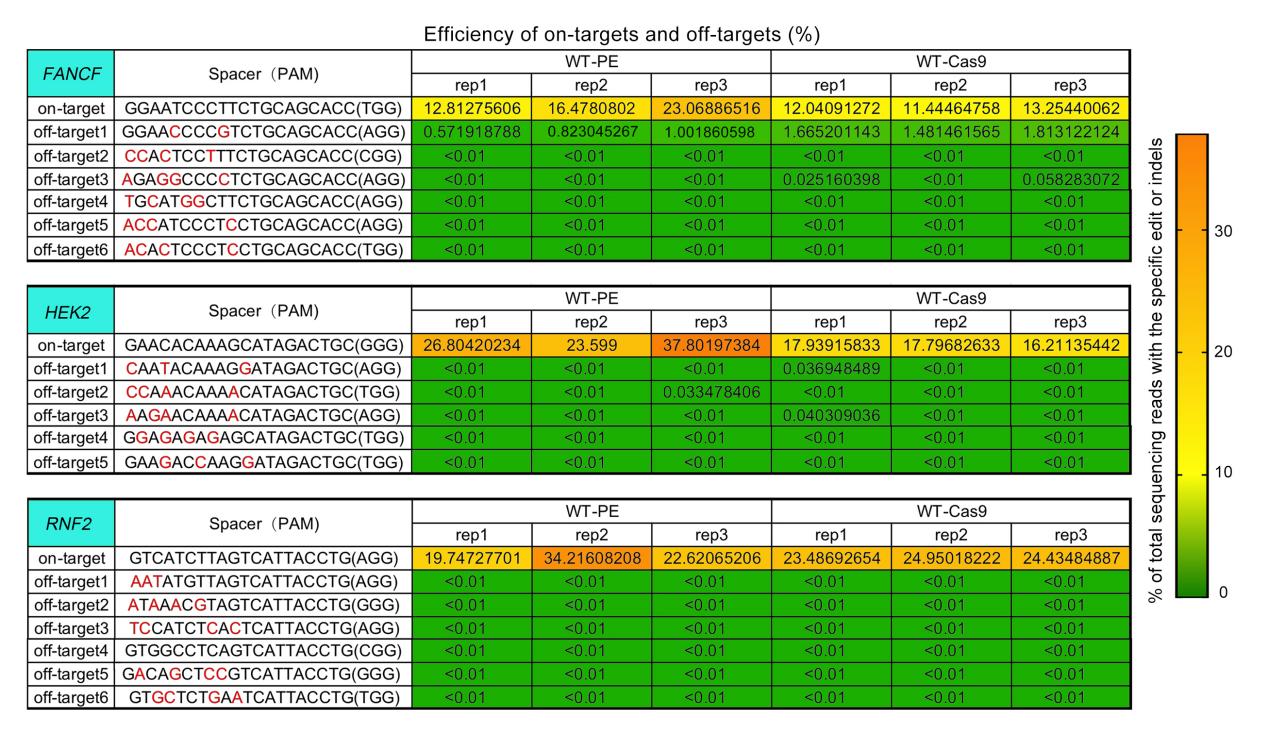


**Figure. S3. Off-target analysis of WT-PE and WT-Cas9 at predicted Cas9 off-target sites.**

NGS analysis of editing outcomes at three on-target and nine off-target sites with indicated editors. For each on-target site, WT-PE was coupled with a pegRNA and WT-Cas9 was coupled with an sgRNA, both of which recognized the same protospacer. For on-target sites, the threshold of editing was set to above 0.2%. The off-target sites corresponding to the three on-target sites were predicted by the CC-TOP website (https://www.genengnews.com/resources/cctop/), with mismatched bases highlighted in red. Rep1, rep2 and rep3 represent three independent biological replicates, and the threshold of editing activity was set to above 0.01%.


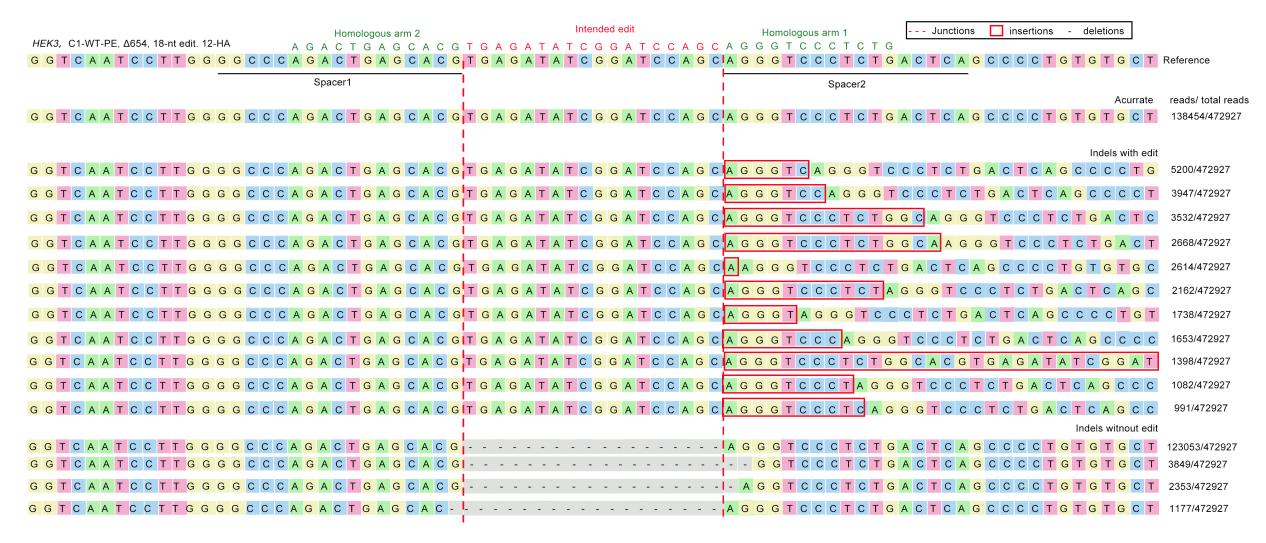


**Figure. S4. Sequence alignment of the *HEK3* alleles edited by C1-WT-PE (Δ654).**

Sequence of the theatrically accurate deletion was used as a reference, with residue spacers underlined. Sequences of the HAs and intended edit were shown on top of the reference. HTS sequencing reads were aligned to the reference sequence and top sequences with a ratio over 0.2% were shown. Vertical dashed lines marked the WT-PE induced DSBs. *HEK3* sequence was used as a reference with sgRNA spacer and PAM sequences underlined. Red box marked HAs encoded by pegRNAs. Horizontal dashed lines indicated deletions.


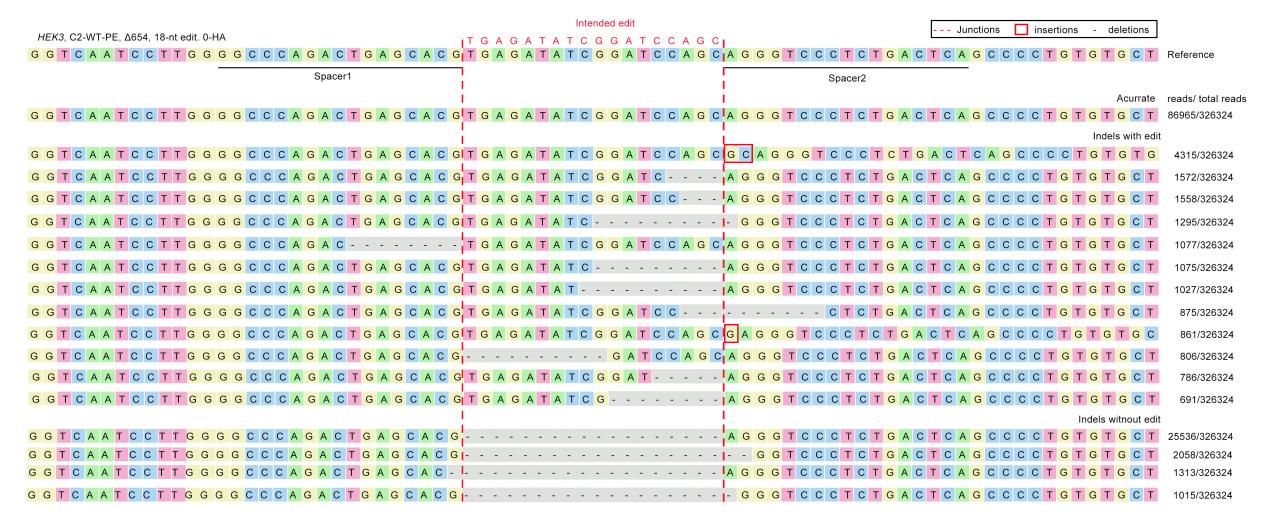


**Figure. S5. Sequence alignment of the *HEK3* alleles edited by C2-WT-PE (Δ654).**

Sequence of the theatrically accurate deletion was used as a reference, with residue spacers underlined. Sequence of the intended edit were shown on top of the reference. HTS sequencing reads were aligned to the reference sequence and top sequences with a ratio over 0.2% were shown. Vertical dashed lines marked the WT-PE induced DSBs. Red box marked the unintended insertions and horizontal dashed lines indicated deletions.


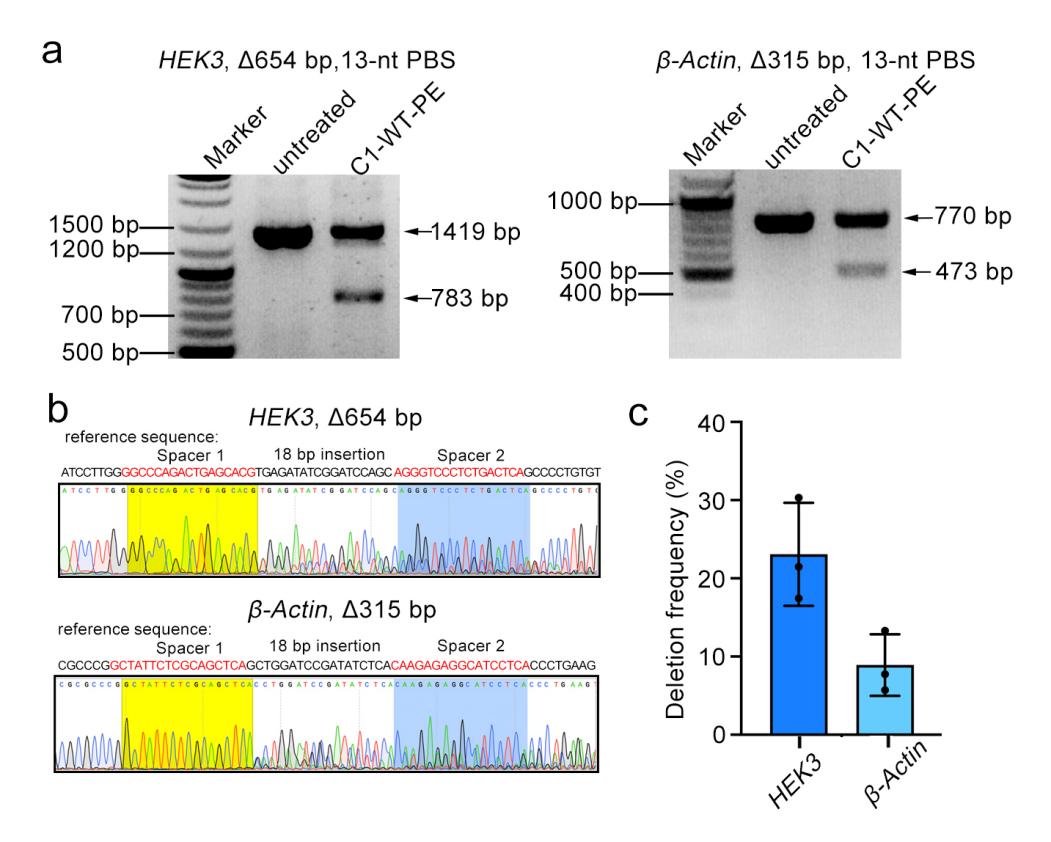


**Figure. S6. Deletion of large genomic fragment in HeLa cells via WT-PE.**

**a.** Agarose gel analysis of the amplicons of targeted deletions. A pair of primers flanking each target deletion were used to amplify the edited region. Bands with size matching wild-type or edited sequences were indicated. **b.** Sanger sequencing chromatograph of each C1-WT-PE edited endogenous site, with reference sequences shown on top. Residue spacer sequences were marked with yellow or blue. **c.** Quantification of the targeted deletion by photoshop software analysis of the band intensity.


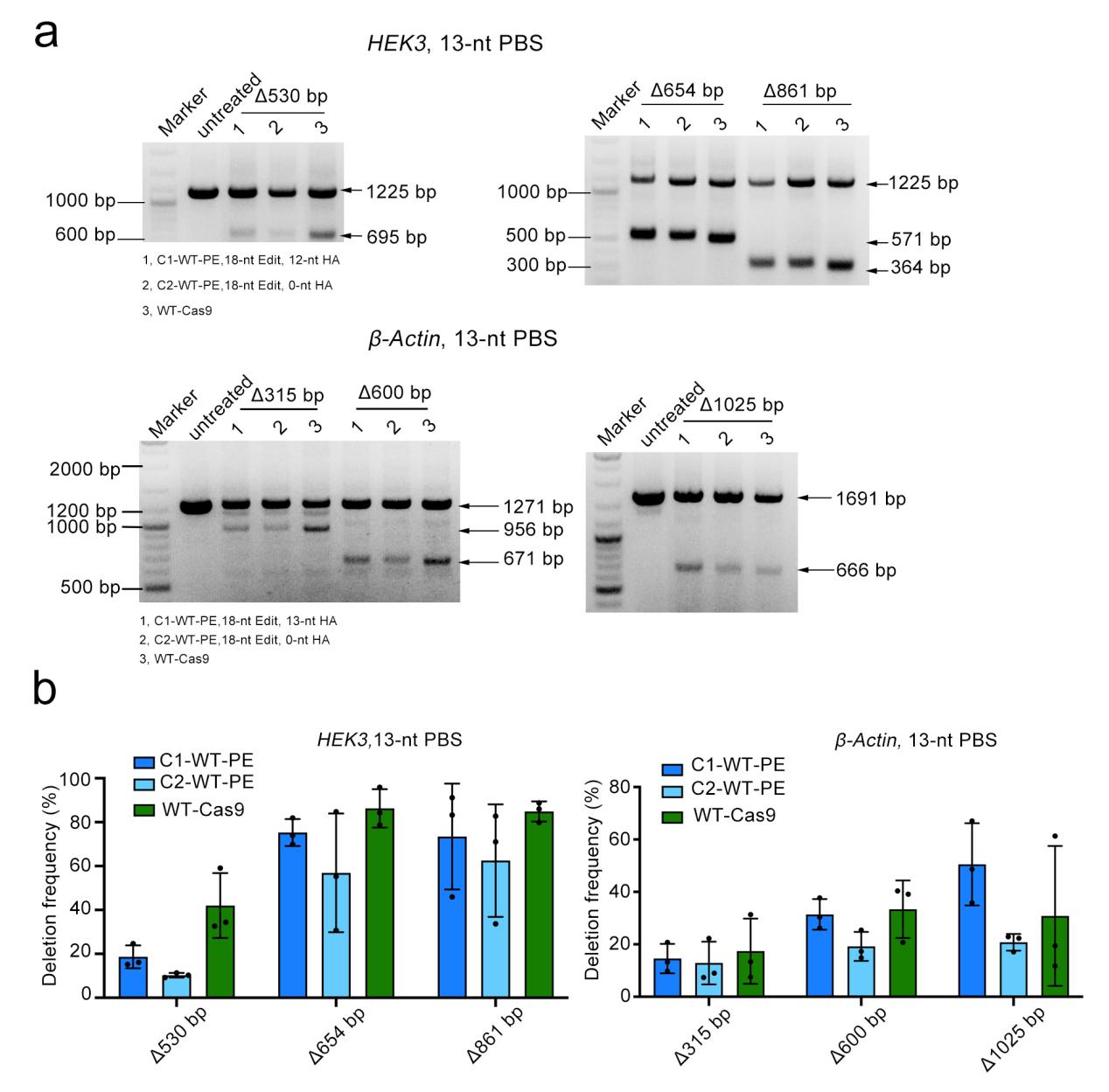


**Figure. S7. Deletion efficiency of WT-PE versus WT-Cas9.**

**a.** Agarose gel analysis of the amplicons of targeted deletions. A pair of primers flanking each target deletion were used to amplify the edited region. Bands with size match wild-type or edited sequences were indicated. Parameters of pegRNA, including the length of edit and HA were indicated below the gel image. **b.** Quantification of the targeted deletion by photoshop software analysis of the band intensity.


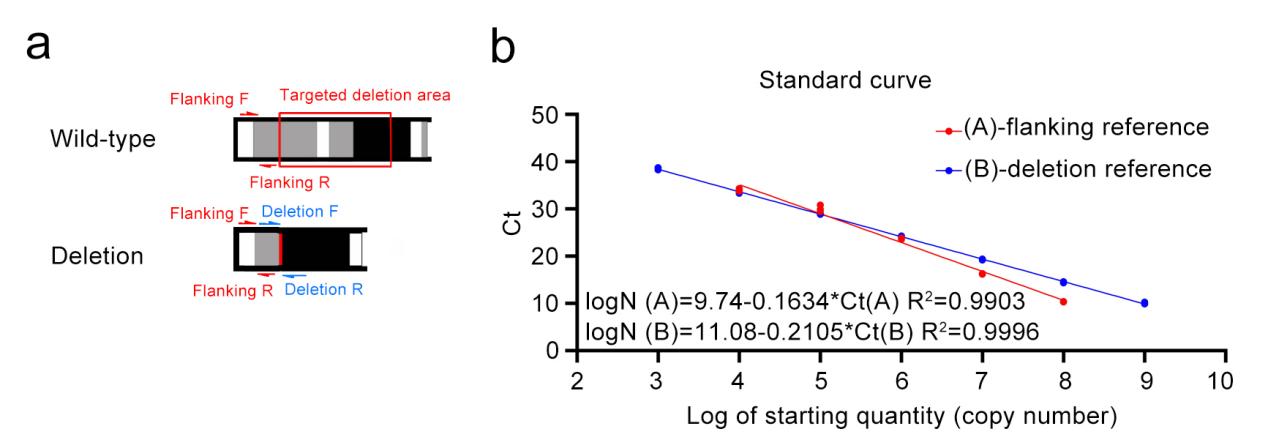


**Figure. S8. Standard curves of absolute quantification of Chr11. deletion.**

**a.** The fragment flanking the target site was used as an internal control reference to indicate total amount of chr.11 and the fragment containing the deletion was used to indicate the amount of chr.11 with deletion. Each fragment was PCR amplified and ligated to the Blunt vector to construct reference plasmid. The primers used to construct the reference plasmids were the same as the qPCR primers (Tables S4); **b.** Stand curves of two reference plasmids. The standard curves for the reference plasmids were determined by CT values against log-transformed concentrations of serial tenfold dilutions (2×10^3^, 10^4^, 10^5^, 10^6^, 10^7^,10^8^ and 10^9^copies per 1μL).


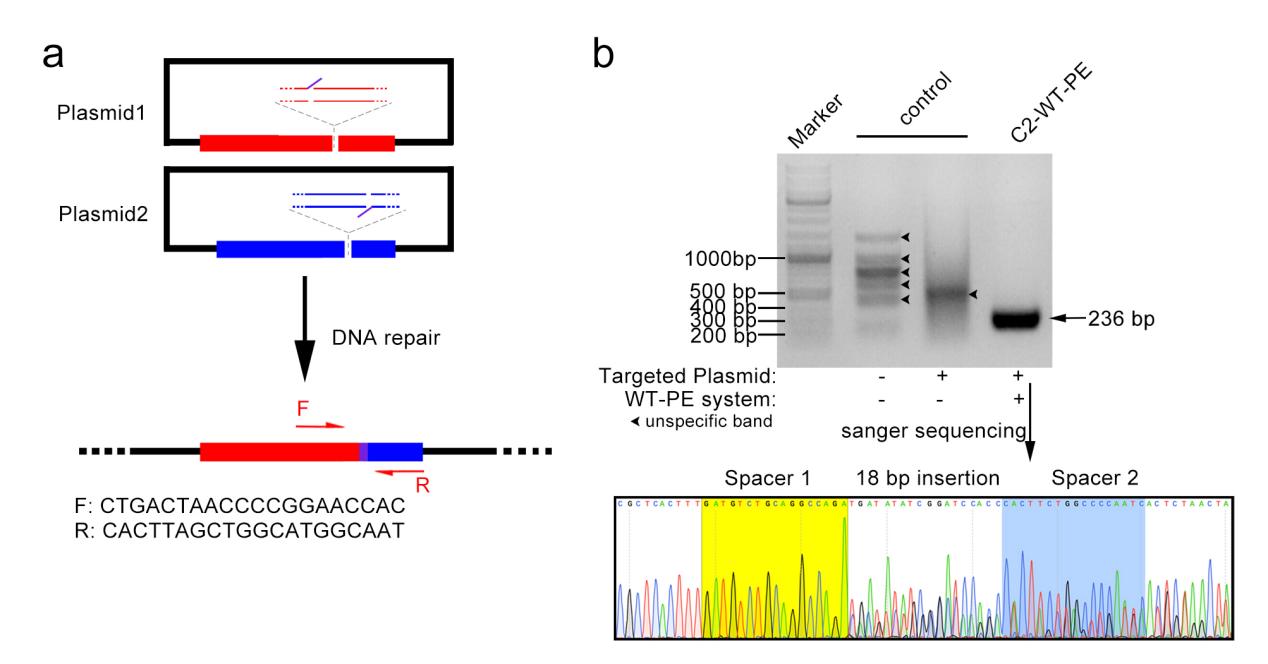


**Figure. S9. Inter-episomal fusion by C2-WT-PE.**

**a.** Diagram showing the design and putative outcome of C2-WT-PE mediated inter-episomal fusion. A pair of primers flanking each end of the prime editing were design to detect the fusion. **b.** The presence of fused plasmids episome was detected by PCR with primers shown in a. Untreated *HEK2*93T cells and same cells transfected with two target plasmids without WT-PE were used as negative control groups. Upper panel showed gel image of the amplicons from each group. Lower panel was Sanger sequencing chromatograph of the amplicons from C2-WT-PE group, with spacers and edit indicated on top of the graph. Primers for PCR analysis were listed in Tables S3.


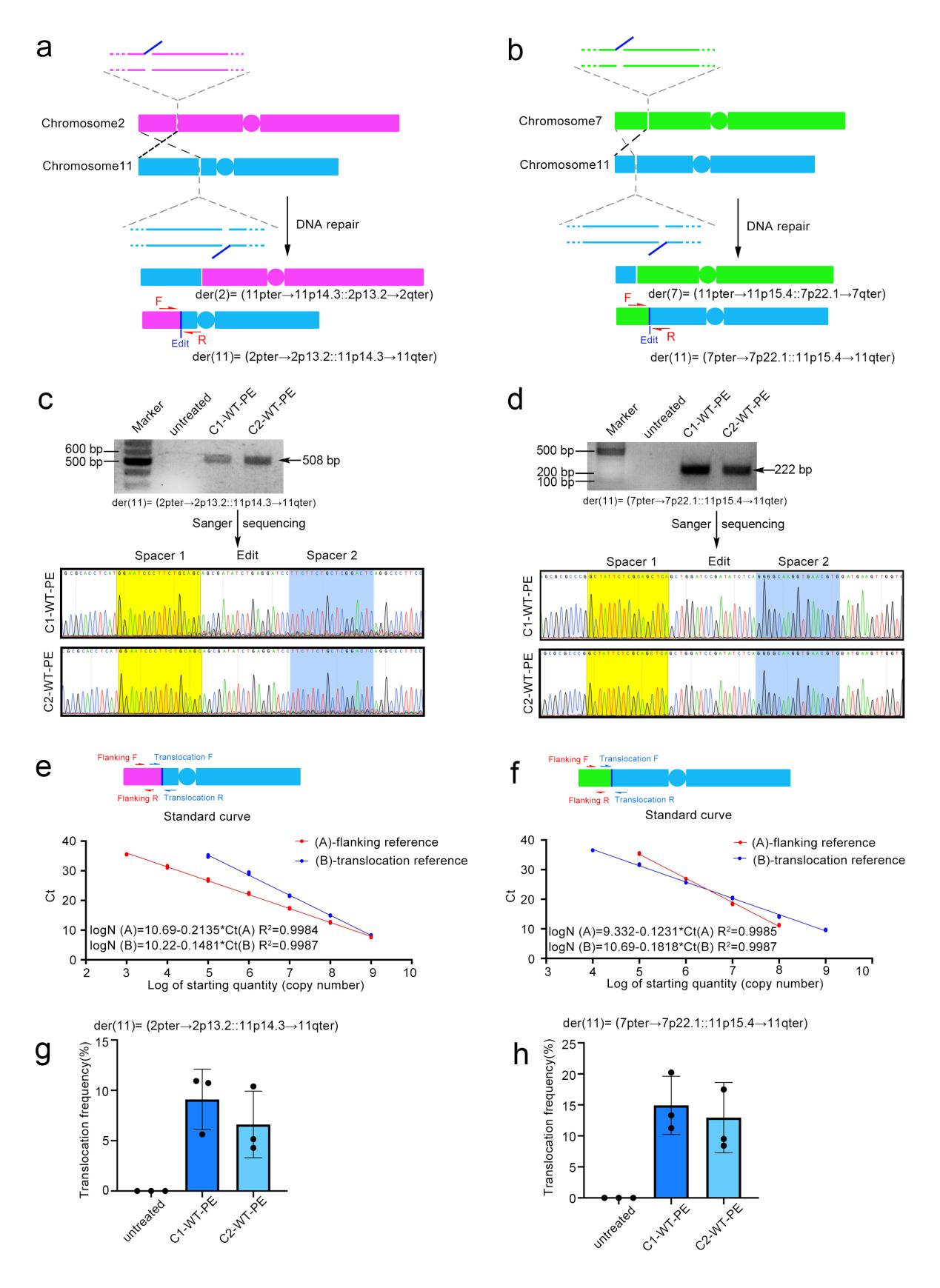


**Figure. S10. Additional targeted inter-chromosomal translocations by WT-PE.**

**a. b.** Diagram showing the design of translocations between chromosomes 2 and 11 and between chromosomes 7 and 11 respectively. The edits that were complementary to each were installed on the small fragment of chromosome 2 or 7 and the large fragment of chromosome 11, which we expected to induce translocations of (**a**) (der11)(2pter→2p13.2::11p14.3→11qter) and (**b**)(der11)( 7pter→7p22.1::11p15.4→11qter). **c. and d.** The presence of the translocation was detected by PCR analysis with primers flanking each side of the translocation. Upper panels showed the agarose gel images of the resulting amplicons and lower panels showed their Sanger sequencing chromatograms with residue spacer sequences marked with yellow or blue. Primers for PCR analysis were listed in Tables S3. **e. and f.** Upper panels showed the schematic diagram of qPCR primer design. The flanking fragment of the target site or the one containing translocation obtained by PCR were ligated to the Blunt vector to construct reference plasmids, The primers used to construct the reference plasmids were the same as the qPCR primers (Tables S4); lower panels showed the standard curves of two reference plasmids. The standard curves for the reference plasmids were determined by CT values against log-transformed concentrations of serial tenfold dilutions (2×10^2^, 10^3^, 10^4^, 10^5^, 10^6^, 10^7^,10^8^ and 10^9^copies per 1μL). **g. and h.** showed percentiles of (der11)(2pter→2p13.2::11p14.3→11qter) and (der11)( 7pter→7p22.1::11p15.4→11qter) translocations respectively.


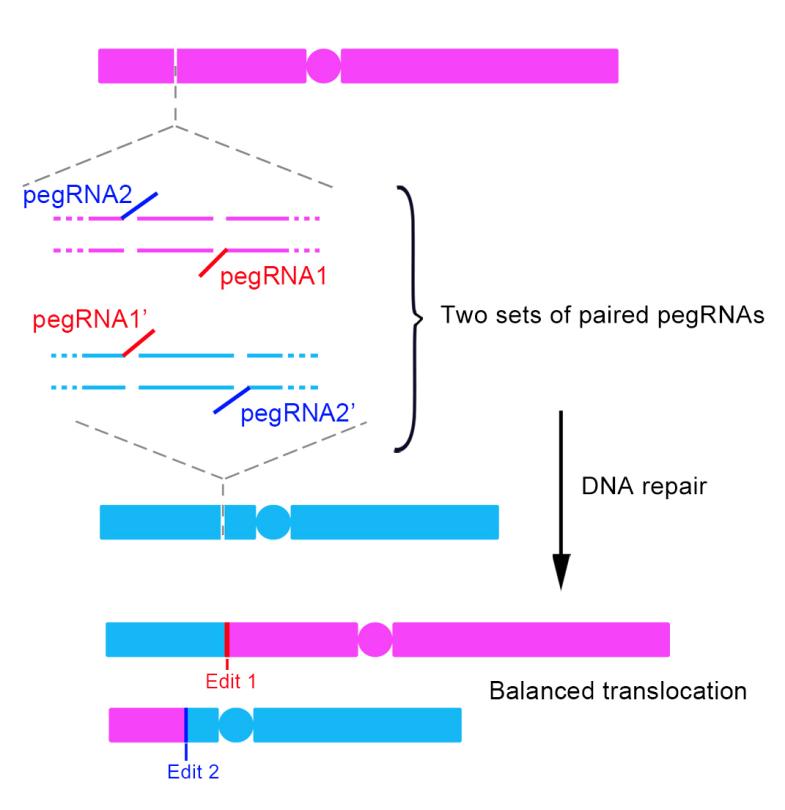


**Figure. S11. WT-PE Strategy for balanced translocation.**


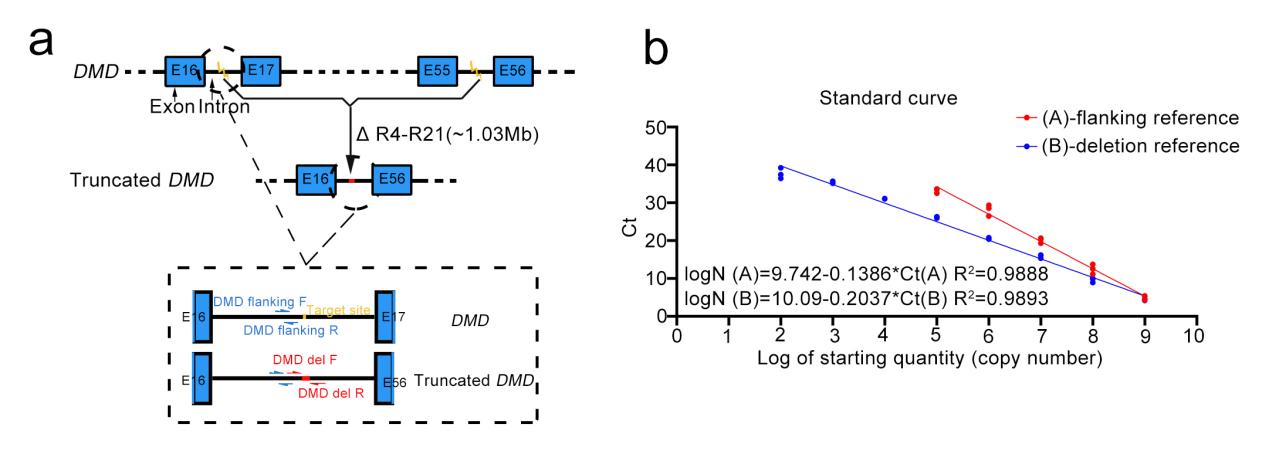


**Figure. S12. Standard curves of absolute quantification of *DMD* deletion.**

**a.** Schematic diagram of qPCR primer design. The flanking fragment of the target site or the one containing deletion obtained by PCR were ligated to the Blunt vector to construct reference plasmids, The primers used to construct the reference plasmids were the same as the qPCR primers (Tables S4); **b.** Standard curves of two reference plasmids. The standard curves for the reference plasmids were determined by CT values against log-transformed concentrations of serial tenfold dilutions (2×10^2^, 10^3^, 10^4^, 10^5^, 10^6^, 10^7^,10^8^ and 10^9^copies per 1μL).


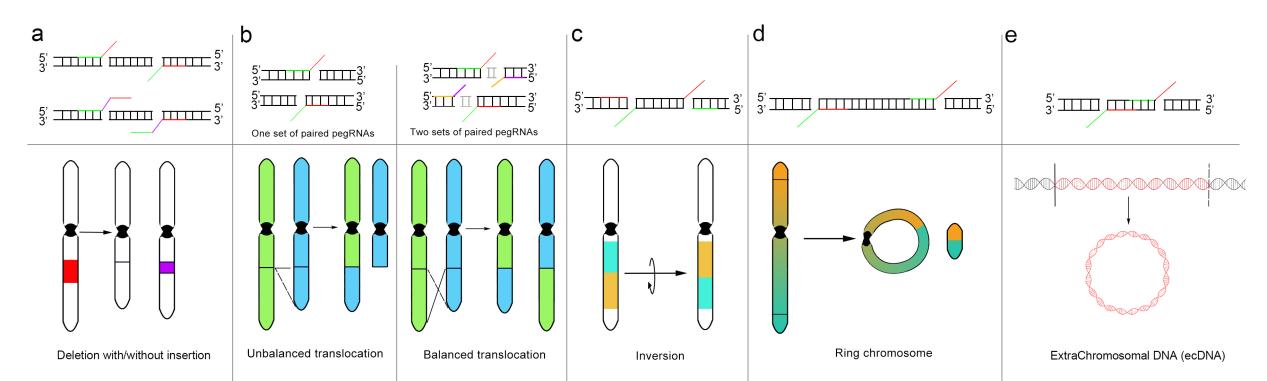


**Figure. S13. Applications of WT-PE in multiple chromosomal manipulations.**

Diagram showing the design of WT-PE mediated large fragment deletion (**a**), unbalanced translocation and balanced translocation (**b**), inversion (**c**), ring chromosome (**d**) and extrachromosomal DNA generation (**e**).

**Tables S1. List of the targets tested in this study.**

| sgRNA | Target sequence **(PAM)** | Oligo-F | Oligo-R | Reference |
| --- | --- | --- | --- | --- |
| *FANCF* | GGAATCCCTTCTGCAGCACC **TGG** | CACCGGAATCCCTTCTGCAGCACC | AAACGGTGCTGCAGAAGGGATTCC | (10) |
| *HEK2* | GAACACAAAGCATAGACTGC **GGG** | CACCGAACACAAAGCATAGACTGC | AAACGCAGTCTATGCTTTGTGTTC | (10) |
| *RNF2* | GTCATCTTAGTCATTACCTG **AGG** | CACCGTCATCTTAGTCATTACCTG | AAACCAGGTAATGACTAAGATGAC | (10) |
| *HEK3*+530 | CCACTTCCAGAGAAGTTGCT **TGG** | CACCGCCACTTCCAGAGAAGTTGCT | AAACAGCAACTTCTCTGGAAGTGGC | This study |
| *HEK3*+654 | TGAGTCAGAGGGACCCTTTG **GGG** | CACCGTGAGTCAGAGGGACCCTTTG | AAACCAAAGGGTCCCTCTGACTCAC | This study |
| *HEK3*+861 | GCTTGGGGCCAGAAGTGTCC **TGG** | CACCGCTTGGGGCCAGAAGTGTCC | AAACGGACACTTCTGGCCCCAAGC | This study |
| *β-Actin* | GCTATTCTCGCAGCTCACCA **TGG** | CACCGCTATTCTCGCAGCTCACCA | AAACTGGTGAGCTGCGAGAATAGC | This study |
| *β-Actin*+315 | TGAGGATGCCTCTCTTGCTC **TGG** | CACCGTGAGGATGCCTCTCTTGCTC | AAACGAGCAAGAGAGGCATCCTCAC | This study |
| *β-Actin*+600 | GGTCAGAGAAGAGAGTCCTA **CGG** | CACCGGTCAGAGAAGAGAGTCCTA | AAACTAGGACTCTCTTCTCTGACC | This study |
| *β-Actin*+1025 | GGAGTCCATCACGATGCCAG **TGG** | CACCGGAGTCCATCACGATGCCAG | AAACCTGGCATCGTGATGGACTCC | This study |
| Chr11.left-deletion | CACGTTCACCTTGCCCCACA **GGG** | CACCGCACGTTCACCTTGCCCCACA | AAACTGTGGGGCAAGGTGAACGTGC | This study |
| Chr11.right-deletion | GGAATCCCTTCTGCAGCACC **TGG** | CACCGGAATCCCTTCTGCAGCACC | AAACGGTGCTGCAGAAGGGATTCC | (10) |
| Chr.6-translocation-1 | GATGTCTGCAGGCCAGATGA **TGG** | CACCGATGTCTGCAGGCCAGATGA | AAACTCATCTGGCCTGCAGACATC | (10) |
| Chr.7-translocation-1 | GCTATTCTCGCAGCTCACCA **TGG** | CACCGCTATTCTCGCAGCTCACCA | AAACTGGTGAGCTGCGAGAATAGC | This study |
| Chr.2-translocation-2 | GAGTCCGAGCAGAAGAAGAA**GGG** | CACCGAGTCCGAGCAGAAGAAGAA | AAACTTCTTCTTCTGCTCGGACTC | (10) |
| Chr.11-translocation-2 | GGAATCCCTTCTGCAGCACC**TGG** | CACCGGAATCCCTTCTGCAGCACC | AAACGGTGCTGCAGAAGGGATTCC | (10) |
| Chr.7-translocation-3 | GCTATTCTCGCAGCTCACCA **TGG** | CACCGCTATTCTCGCAGCTCACCA | AAACTGGTGAGCTGCGAGAATAGC | This study |
| Chr.11-translocation-3 | CACGTTCACCTTGCCCCACA  **GGG** | CACCGCACGTTCACCTTGCCCCACA | AAACTGTGGGGCAAGGTGAACGTGC | This study |
| *DMD* E16-17 | GGAGGAGTAGAAGTGATGGT **GGG** | CACCGGAGGAGTAGAAGTGATGGT | AAACACCATCACTTCTACTCCTCC | This study |
| *DMD* E55-56 | ACTAAAAGCTTAGTTTACAG **AGG** | CACCGACTAAAAGCTTAGTTTACAG | AAACCTGTAAACTAAGCTTTTAGTC | This study |

**Tables S2. List of the off-targets tested in this study.**

| Off-target sites | Off-target sequence **(PAM)** |
| --- | --- |
| FANCF-OFF1 | TCATTCCCGTCTGCAGCACC**(CGG)** |
| FANCF-OFF2 | CCACTCCTTTCTGCAGCACC**(CGG)** |
| FANCF-OFF3 | AGAGGCCCCTCTGCAGCACC**(AGG)** |
| FANCF-OFF4 | TGCATGGCTTCTGCAGCACC**(CGG)** |
| FANCF-OFF5 | ACCATCCCTCCTGCAGCACC**(AGG)** |
| FANCF-OFF6 | ACACTCCCTCCTGCAGCACC**(TGG)** |
| HEK2-OFF1 | CAATACAAAGGATAGACTGC**(AGG)** |
| HEK2-OFF2 | CCAAACAAAACATAGACTGC**(TGG)** |
| HEK2-OFF3 | AAGAACAAAACATAGACTGC**(AGG)** |
| HEK2-OFF4 | GGAGAGAGAGCATAGACTGC**(TGG)** |
| HEK2-OFF5 | GAAGACCAAGGATAGACTGC**(TGG)** |
| RNF2-OFF1 | AATATGTTAGTCATTACCTG**(AGG)** |
| RNF2-OFF2 | ATAAACGTAGTCATTACCTG**(GGG)** |
| RNF2-OFF3 | TCCATCTCACTCATTACCTG**(AGG)** |
| RNF2-OFF4 | GTGGCCTCAGTCATTACCTG**(CGG)** |
| RNF2-OFF5 | GACAGCTCCGTCATTACCTG**(GGG)** |
| RNF2-OFF6 | GTGCTCTGAATCATTACCTG**(TGG)** |

**Tables S3. Sequences of pegRNAs used for in vitro experiments.**

| pegRNA | Spacer | 3' extension (5' to 3') | PBS length  (nt) | HA  Length (nt) | Edit  (nt) |
| --- | --- | --- | --- | --- | --- |
| *FANCF*+5 G-T peg | GGAATCCCTTCTGCAGCACC | GGAAAAGCGATCAAGGTGCTGCAGAAGGGATT | 15 | 12 | 5 |
| *HEK2* +1 T -A peg | GAACACAAAGCATAGACTGC | CTGGCCCGCCCCGCTGTCTATGCTTTGTGT | 15 | 14 | 1 |
| *RNF2* +1 C-A peg | GTCATCTTAGTCATTACCTG | AACGAACACCTCATGTAATGACTAAGATG | 15 | 13 | 1 |
| C1-*HEK3*-Δ530 peg | GGCCCAGACTGAGCACGTGA | TCCAGAGAAGTTGCTGGATCCGATATCTCACGTGCTCAGTCTG | 13 | 12 | 18 |
| C1-*HEK3*-Δ654 peg | GGCCCAGACTGAGCACGTGA | CAGAGGGACCCTGCTGGATCCGATATCTCACGTGCTCAGTCTG | 13 | 12 | 18 |
| C1-*HEK3*-Δ861 peg | GGCCCAGACTGAGCACGTGA | GGGCCAGAAGTGGCTGGATCCGATATCTCACGTGCTCAGTCTG | 13 | 12 | 18 |
| C1-*HEK3* +530 peg | CCACTTCCAGAGAAGTTGCT | AGACTGAGCACGTGAGATATCGGATCCAGCAACTTCTCTGGAA | 13 | 12 | 18 |
| C1-*HEK3* +654 peg | TGAGTCAGAGGGACCCTTTG | AGACTGAGCACGTGAGATATCGGATCCAGCAGGGTCCCTCTGA | 13 | 12 | 18 |
| C1-*HEK3* +861 peg | GCTTGGGGCCAGAAGTGTCC | AGACTGAGCACGTGAGATATCGGATCCAGCCACTTCTGGCCCC | 13 | 12 | 18 |
| C2-*HEK3*-peg | GGCCCAGACTGAGCACGTGA | GCTGGATCCGATATCTCACGTGCTCAGTCTG | 13 | 0 | 18 |
| C2-*HEK3* +530 peg | CCACTTCCAGAGAAGTTGCT | TGAGATATCGGATCCAGCAACTTCTCTGGAA | 13 | 0 | 18 |
| C2-*HEK3* +654 peg | TGAGTCAGAGGGACCCTTTG | TGAGATATCGGATCCAGCAGGGTCCCTCTGA | 13 | 0 | 18 |
| C2-*HEK3* +861 peg | GCTTGGGGCCAGAAGTGTCC | TGAGATATCGGATCCAGCCACTTCTGGCCCC | 13 | 0 | 18 |
| C1-*β-Actin*-Δ315 peg | GCTATTCTCGCAGCTCACCA | GATGCCTCTCTTGTGAGATATCGGATCCAGCTGAGCTGCGAGAA | 13 | 13 | 18 |
| C1-*β-Actin*-Δ600 peg | GCTATTCTCGCAGCTCACCA | AGAGAAGAGAGTCTGAGATATCGGATCCAGCTGAGCTGCGAGAA | 13 | 13 | 18 |
| C1-*β-Actin*-Δ1025 peg | GCTATTCTCGCAGCTCACCA | TCCATCACGATGCTGAGATATCGGATCCAGCTGAGCTGCGAGAA | 13 | 13 | 18 |
| C1-*β-Actin* +315 peg | TGAGGATGCCTCTCTTGCTC | TCTCGCAGCTCAGCTGGATCCGATATCTCACAAGAGAGGCATCCT | 15 | 12 | 18 |
| C1-*β-Actin* +600 peg | GGTCAGAGAAGAGAGTCCTA | TCTCGCAGCTCAGCTGGATCCGATATCTCAGACTCTCTTCTCTGA | 15 | 12 | 18 |
| C1-*β-Actin* +1025 peg | GGAGTCCATCACGATGCCAG | TCTCGCAGCTCAGCTGGATCCGATATCTCAGCATCGTGATGGA | 13 | 12 | 18 |
| C2-*β-Actin* E18 peg | GCTATTCTCGCAGCTCACCA | GCTGGATCCGATATCTCATGAGCTGCGAGAA | 13 | 0 | 18 |
| C2*β-Actin* +315 peg | TGAGGATGCCTCTCTTGCTC | GCTGGATCCGATATCTCACAAGAGAGGCATCCT | 15 | 0 | 18 |
| C2-*β-Actin* +600 peg | GGTCAGAGAAGAGAGTCCTA | GCTGGATCCGATATCTCAGACTCTCTTCTCTGA | 15 | 0 | 18 |
| C2-*β-Actin*+1025 peg | GGAGTCCATCACGATGCCAG | GCTGGATCCGATATCTCAGCATCGTGATGGA | 13 | 0 | 18 |
| C1-Chr.11 left peg | CACGTTCACCTTGCCCCACA | GTGGCGGGGTCCCAGGTGCTGAGCTGGATCCGATATCTCA GGGGCAAGGTGAAC | 14 | 22 | 18 |
| C1-Chr.11 right peg | GGAATCCCTTCTGCAGCACC | TCATCCACGTTCACCTTGCCCCTGAGATATCGGATCCAGCGCTGCAGAAGGGAT | 14 | 22 | 18 |
| C2-Chr.11 left peg | CACGTTCACCTTGCCCCACA | GCTGGATCCGATATCTCA GGGGCAAGGTGAAC | 14 | 0 | 18 |
| C2-Chr.11 right peg | GGAATCCCTTCTGCAGCACC | TGAGATATCGGATCCAGCGCTGCAGAAGGGAT | 14 | 0 | 18 |
| C2-Plasmid 1-peg | GCTTGGGGCCAGAAGTGTCC | TGAGATATCGGATCCAGCCACTTCTGGCCCC | 13 | 0 | 18 |
| C2-Plasmid 2-peg | GATGTCTGCAGGCCAGATGA | GCTGGATCCGATATCTCATCTGGCCTGCAGA | 13 | 0 | 18 |
| C1-Translocation-Chr.6 peg-1 | GATGTCTGCAGGCCAGATGA | GACCTCGGCTCACAGCGCGCCCGGCTATTCTCGCAGCTCAGCTGGATCCGATATCTCATCTGGCCTGCAGA | 13 | 40 | 18 |
| C1-Translocation-Chr.7 peg-1 | GCTATTCTCGCAGCTCACCA | GCCTGTTGGCTGCCGCTCACTTTGATGTCTGCAGGCCAGATGAGATATCGGATCCAGCTGAGCTGCGAGAA | 13 | 40 | 18 |
| C2-Translocation-Chr.6 peg-1 | GATGTCTGCAGGCCAGATGA | GCTGGATCCGATATCTCATCTGGCCTGCAGA | 13 | 0 | 18 |
| C2-Translocation-Chr.7 peg-1 | GCTATTCTCGCAGCTCACCA | TGAGATATCGGATCCAGCTGAGCTGCGAGAA | 13 | 0 | 18 |
| C1-Translocation-Chr.2 peg-2 | GAGTCCGAGCAGAAGAAGAA | ACCTCATGGAATCCCTTCTGCAGCAGCGATATCTGAGGATCC TTCTTCTGCTCGG | 13 | 24 | 18 |
| C1-Translocation-Chr.11 peg-2 | GGAATCCCTTCTGCAGCACC | AAGGGCCTGAGTCCGAGCAGAAGAAGGATCCTCAGATATCGCTGCTGCAGAAGGGA | 13 | 25 | 18 |
| C2-Translocation-Chr.2 peg-2 | GAGTCCGAGCAGAAGAAGAA | AGCGATATCTGAGGATCCTTCTTCTGCTCGG | 13 | 0 | 18 |
| C2-Translocation-Chr.11 peg-2 | GGAATCCCTTCTGCAGCACC | GGATCCTCAGATATCGCTGCTGCAGAAGGGA | 13 | 0 | 18 |
| C1-Translocation-Chr.7 peg-3 | GCTATTCTCGCAGCTCACCA | TCACCACCAACTTCATCCACGTTCACCTTGCCCCTGAGATATCGGATCCAGC TGAGCTGCGAGAA | 13 | 34 | 18 |
| C1-Translocation-Chr.11 peg-3 | CACGTTCACCTTGCCCCACA | GGCTCACAGCGCGCCCGGCTATTCTCGCAGCTCAGCTGGATCCGATATCTCA GGGGCAAGGTGAAC | 14 | 34 | 18 |
| C2-Translocation-Chr.7 peg-3 | GCTATTCTCGCAGCTCACCA | TGAGATATCGGATCCAGCTGAGCTGCGAGAA | 13 | 0 | 18 |
| C2-Translocation-Chr.11 peg-3 | CACGTTCACCTTGCCCCACA | GCTGGATCCGATATCTCAGGGGCAAGGTGAAC | 14 | 0 | 18 |
| C1-*DMD* E16-17 peg | GGAGGAGTAGAAGTGATGGT | ACTACAACTAAAAGCTTAGTTTAGCTGGATCCGATATCTCAATCACTTCTACTC | 13 | 23 | 18 |
| C1-*DMD* E55-56 peg | ACTAAAAGCTTAGTTTACAG | TGGGTAGGAGGAGTAGAAGTGATTGAGATATCGGATCCAGCTAAACTAAGCTTT | 13 | 23 | 18 |
| C2-*DMD* E16-17 peg | GGAGGAGTAGAAGTGATGGT | GCTGGATCCGATATCTCAATCACTTCTACTC | 13 | 0 | 18 |
| C2-*DMD* E55-56 peg | ACTAAAAGCTTAGTTTACAG | TGAGATATCGGATCCAGCTAAACTAAGCTTT | 13 | 0 | 18 |

**Tables S4. Summary of primers for amplification of each target sites.**

| Target site | Length (bps) | Forward primer | Reverse primer | Figures |
| --- | --- | --- | --- | --- |
| *HEK3* | 1225 | TGATGTGGGCTGCCTAGAAA | AAATCCTCGCATTTGGGCAG | Figure 2b |
| *β-Actin* | 1691 | CTGTGTTGGCGTACAGGTCT | GGCTTCCTTTGTCCCCAATC | Figure 2b |
| *β-Actin* | 1271 | GAACACGGCTAAGTGTGCTG | GACCCGGCGCTGTTTGAAC | Figure 2b |
| Chr11. deletion | 491 | GTAGACCACCAGCAGCCTAA | CGTGGTTCCGGAAATTCTCG | Figure 2e, f |
| Chr6. and Chr7.  translocation | 187 | CTTCCCAAAGGACCCCAGTC | CGCGCGCCGGCGCCCCCTGG | Figure 3b |
| *DMD* deletion | 631 | GTTTTCCCATCCTCACCTGC | CTTTCATTCCCCGCCCTTG | Figure 4c |
| Site1 | 500 | TCACAGTGGCAAATGAGGC | CCTCTGTTGAGCTCGACCCT | Supplementary Figure 2b |
| Site2 | 450 | CCCCAAAGGGTCCCTCTGAC | TCAGTTCTGTGAACCTGTAT | Supplementary Figure 2b |
| Site3 | 629 | AGTCTGGGCTTGGGCTGATA | GAATGGGCTTTGGAAAGGGG | Supplementary Figure 2b |
| *HEK3* (Hela) | 1419 | TCACAGTGGCAAATGAGGCT | AAGGGTTTGGGCTTCGGATA | Supplementary Figure 5a |
| *β-Actin* (Hela) | 770 | GGCTTCCTTTGTCCCCAATCTG | ACCTGCAGAGTTCCAAAGGA | Supplementary Figure 5a |
| Inter-episomal | 236 | CTGACTAACCCCGGAACCAC | ATTGCCATGCCAGCTAAGTG | Supplementary Figure 9b |
| Chr2. and Chr11.  translocation | 508 | GCCCCTAACCCTATGTAGCC | CGTGGTTCCGGAAATTCTCG | Supplementary Figure 10c |
| Chr7. and Chr11.  translocation | 222 | CTCACCACCAACTTCATCCACG | CCGACCAGTGTTTGCCTTTT | Supplementary Figure 10d |

**Tables S5. Primers used for qPCR to detect the copy number of target DNA fragments.**

| Target site | Forward primer | Reverse primer |
| --- | --- | --- |
| Chr11. flanking | TCTCCACATGCCCAGTTTCT | CTGGGCAGGTTGGTATCAA |
| Chr11. deletion | GCCTCACCACCAACTTCATC | ATGGATGTGGCGCAGGTAG |
| *DMD* flanking | CCACTCTCAAATAGGCCCCA | TGAGAATACTGGAATCTGAGGCA |
| *DMD* deletion | TGTGGTGGGTAGGAGGAGTA | CAAGAACTTCCCTCTGCCCT |
| Chr2. and Chr11.  Translocation- flanking | GCCATCCCCTTCTGTGAAT | AGGGGCACAGATGAGAAACT |
| Chr2. and Chr11.  Translocation-  translocation | AGTTTCTCATCTGTGCCCCT | ATCAGTACGCAGAGAGTCGC |
| Chr7. and Chr11.  Translocation- flanking | TCTCCACATGCCCAGTTTCT | CTGGGCAGGTTGGTATCAA |
| Chr7. and Chr11.  Translocation-  translocation | CTCACCACCAACTTCATCCACG | CTGGCGGCCTAAGGACTC |

**Tables S6. HTS primers used for mammalian cell genomic DNA amplification.**

| Sample name | Primer Name | Sequence |
| --- | --- | --- |
| *FANCF*+5G-T | *FANCF*-HTS -1-few | acgttctTGCATTTGTAGGCTTGATGC |
|  | *FANCF*-HTS -rev | AGGTAGCGCGCCCACTGCAA |
| *FANCF*+5G-T | *FANCF*-HTS -2-few | accggttTCCAATCAGTACGCAGAGAG |
|  | *FANCF*-HTS -rev | AGGTAGCGCGCCCACTGCAA |
| *FANCF*+5G-T | *FANCF*-HTS -2-few | agacatgTCCAATCAGTACGCAGAGAG |
|  | *FANCF*-HTS -rev | AGGTAGCGCGCCCACTGCAA |
| Untreated-*FANCF*+5G-T | *FANCF*-HTS -4-few | actacgtTCCAATCAGTACGCAGAGAG |
|  | *FANCF*-HTS -rev | AGGTAGCGCGCCCACTGCAA |
| *HEK2*+1T-A | *HEK2*-HTS -1-few | tgagcagTGCATTTGTAGGCTTGATGC |
|  | *HEK2*-HTS -rev | TGTCCAGCCCCATCTGTCAA |
| *HEK2*+1T-A | *HEK2*-HTS -2-few | actcaagTGTATCCCAGTTTACACGTCTCA |
|  | *HEK2*-HTS -rev | TGTCCAGCCCCATCTGTCAA |
| *HEK2*+1T-A | *HEK2*-HTS -3-few | acagcatTGATAACAAGACCTGGCTGA |
|  | *HEK2*-HTS -rev | TGTCCAGCCCCATCTGTCAA |
| Untreated-*HEK2*+1T-A | *HEK2*-HTS -4-few | actgttgTGATAACAAGACCTGGCTGA |
|  | *HEK2*-HTS -rev | TGTCCAGCCCCATCTGTCAA |
| *RNF2*+1C-A | *RNF2*-HTS -1-few | actagacTGTATCCCAGTTTACACGTCTCA |
|  | *RNF2*-HTS -rev | TTTCCAGCAATGTCTCAGGC |
| *RNF2*+1C-A | *RNF2*-HTS -2-few | acgtctcTGTATCCCAGTTTACACGTCTCA |
|  | *RNF2*-HTS -rev | TTTCCAGCAATGTCTCAGGC |
| *RNF2*+1C-A | *RNF2*-HTS -3-few | actcaagTGTATCCCAGTTTACACGTCTCA |
|  | *RNF2*-HTS -rev | TTTCCAGCAATGTCTCAGGC |
| Untreated-*RNF2*+1C-A | *RNF2*-HTS -4-few | acttgcaTGTATCCCAGTTTACACGTCTCA |
|  | *RNF2*-HTS --rev | TTTCCAGCAATGTCTCAGGC |
| C2-*HEK3*-Δ530 bp | *HEK3*-HTS -1-few | acgttctTGCATTTGTAGGCTTGATGC |
|  | *HEK3*-HTS -530-rev | TGGCTTTAACCCTCCAGTGT |
| C2-*HEK3*-Δ530 bp | *HEK3*-HTS -2-few | tgacaacTGCATTTGTAGGCTTGATGC |
|  | *HEK3*-HTS -530-rev | TGGCTTTAACCCTCCAGTGT |
| C2-*HEK3*-Δ530 bp | *HEK3*-HTS -3-few | tgttgacTGCATTTGTAGGCTTGATGC |
|  | *HEK3*-HTS -530-rev | TGGCTTTAACCCTCCAGTGT |
| C1-*HEK3*-Δ530 bp | *HEK3*-HTS -4-few | agaagtcTGCATTTGTAGGCTTGATGC |
|  | *HEK3*-HTS -530-rev | TGGCTTTAACCCTCCAGTGT |
| C1-*HEK3*-Δ530 bp | *HEK3*-HTS -5-few | tgagcagTGCATTTGTAGGCTTGATGC |
|  | *HEK3*-HTS -530-rev | TGGCTTTAACCCTCCAGTGT |
| C1-*HEK3*-Δ530 bp | *HEK3*-HTS -6-few | tgcagttTGCATTTGTAGGCTTGATGC |
|  | *HEK3*-HTS -530-rev | TGGCTTTAACCCTCCAGTGT |
| C2-*HEK3*-Δ654 bp | *HEK3*-HTS -7-few | caagtagTGCATTTGTAGGCTTGATGC |
|  | *HEK3*-HTS -654-rev | TTCCAGCTCTAGAAGGCCAC |
| C2-*HEK3*-Δ654 bp | *HEK3*-HTS -8-few | gagcgatTGCATTTGTAGGCTTGATGC |
|  | *HEK3*-HTS -654-rev | TTCCAGCTCTAGAAGGCCAC |
| C2-*HEK3*-Δ654 bp | *HEK3*-HTS -9-few | gttcaacTGCATTTGTAGGCTTGATGC |
|  | *HEK3*-HTS -654-rev | TTCCAGCTCTAGAAGGCCAC |
| C1-*HEK3*-Δ654 bp | *HEK3*-HTS -10-few | catcttgTGCATTTGTAGGCTTGATGC |
|  | *HEK3*-HTS -654-rev | TTCCAGCTCTAGAAGGCCAC |
| C1-*HEK3*-Δ654 bp | *HEK3*-HTS -11-few | gtatgcaTGCATTTGTAGGCTTGATGC |
|  | *HEK3*-HTS -654-rev | TTCCAGCTCTAGAAGGCCAC |
| C1-*HEK3*-Δ654 bp | *HEK3*-HTS -12-few | gttcgtaTGCATTTGTAGGCTTGATGC |
|  | *HEK3*-HTS -654-rev | TTCCAGCTCTAGAAGGCCAC |
| C2-*HEK3*-Δ861bp | *HEK3*-HTS -13-few | gcgagttTGCATTTGTAGGCTTGATGC |
|  | *HEK3*-HTS -861-rev | CACTTAGCTGGCATGGCAAT |
| C2-*HEK3*-Δ861bp | *HEK3*-HTS -14-few | aactaggTGCATTTGTAGGCTTGATGC |
|  | *HEK3*-HTS -861-rev | CACTTAGCTGGCATGGCAAT |
| C2-*HEK3*-Δ861bp | *HEK3*-HTS -15-few | aagatgcTGCATTTGTAGGCTTGATGC |
|  | *HEK3*-HTS -861-rev | CACTTAGCTGGCATGGCAAT |
| C1-*HEK3*-Δ861bp | *HEK3*-HTS -16-few | acacagtTGCATTTGTAGGCTTGATGC |
|  | *HEK3*-HTS -861-rev | CACTTAGCTGGCATGGCAAT |
| C1-*HEK3*-Δ861bp | *HEK3*-HTS -17-few | acagtcaTGCATTTGTAGGCTTGATGC |
|  | *HEK3*-HTS -861-rev | CACTTAGCTGGCATGGCAAT |
| C1-*HEK3*-Δ861bp | *HEK3*-HTS -18-few | actctgaTGCATTTGTAGGCTTGATGC |
|  | *HEK3*-HTS -861-rev | CACTTAGCTGGCATGGCAAT |
| C2-*β-Actin*-Δ315 bp | *β-Actin*-HTS -1-few | gacagtaGGCTTCCTTTGTCCCCAATC |
|  | *β-Actin*-HTS -315-rev | TTGTAGAAGGTGTGGTGCCA |
| C2-*β-Actin*-Δ315 bp | *β-Actin*-HTS -2-few | gactacaGGCTTCCTTTGTCCCCAATC |
|  | *β-Actin*-HTS -315-rev | TTGTAGAAGGTGTGGTGCCA |
| C2-*β-Actin*-Δ315 bp | *β-Actin*-HTS -3-few | gacagtaGGCTTCCTTTGTCCCCAATC |
|  | *β-Actin*-HTS -315-rev | TTGTAGAAGGTGTGGTGCCA |
| C1-*β-Actin*-Δ315 bp | *β-Actin*-HTS -4-few | gactacaGGCTTCCTTTGTCCCCAATC |
|  | *β-Actin*-HTS -315-rev | TTGTAGAAGGTGTGGTGCCA |
| C1-*β-Actin*-Δ315 bp | *β-Actin*-HTS -5-few | gacagtaGGCTTCCTTTGTCCCCAATC |
|  | *β-Actin*-HTS -315-rev | TTGTAGAAGGTGTGGTGCCA |
| C1-*β-Actin*-Δ315 bp | *β-Actin*-HTS -6-few | gactacaGGCTTCCTTTGTCCCCAATC |
|  | *β-Actin*-HTS -315-rev | TTGTAGAAGGTGTGGTGCCA |
| C2-*β-Actin*-Δ600 bp | *β-Actin*-HTS -7-few | acttagcGGCTTCCTTTGTCCCCAATC |
|  | *β-Actin*-HTS -600-rev | ACCTGCAGAGTTCCAAAGGA |
| C2-*β-Actin*-Δ600 bp | *β-Actin*-HTS -8-few | ctgatgtGGCTTCCTTTGTCCCCAATC |
|  | *β-Actin*-HTS -600-rev | ACCTGCAGAGTTCCAAAGGA |
| C2-*β-Actin*-Δ600 bp | *β-Actin*-HTS -9-few | ctgtagaGGCTTCCTTTGTCCCCAATC |
|  | *β-Actin*-HTS -600-rev | ACCTGCAGAGTTCCAAAGGA |
| C1-*β-Actin*-Δ600 bp | *β-Actin*-HTS -10-few | gaacactGGCTTCCTTTGTCCCCAATC |
|  | *β-Actin*-HTS -600-rev | ACCTGCAGAGTTCCAAAGGA |
| C1-*β-Actin*-Δ600 bp | *β-Actin*-HTS -11-few | gatctcaGGCTTCCTTTGTCCCCAATC |
|  | *β-Actin*-HTS -600-rev | ACCTGCAGAGTTCCAAAGGA |
| C1-*β-Actin*-Δ600 bp | *β-Actin*-HTS -12-few | gacatctGGCTTCCTTTGTCCCCAATC |
|  | *β-Actin*-HTS -600-rev | ACCTGCAGAGTTCCAAAGGA |
| C2-*β-Actin*-Δ1025 bp | *β-Actin*-HTS -13-few | atcaagcGGCTTCCTTTGTCCCCAATC |
|  | *β-Actin*-HTS -1025-rev | GAGGTAGTCAGTCAGGTCCC |
| C2-*β-Actin*-Δ1025 bp | *β-Actin*-HTS -14-few | atccggaGGCTTCCTTTGTCCCCAATC |
|  | *β-Actin*-HTS -1025-rev | GAGGTAGTCAGTCAGGTCCC |
| C2-*β-Actin*-Δ1025 bp | *β-Actin*-HTS -15-few | atcgaagGGCTTCCTTTGTCCCCAATC |
|  | *β-Actin*-HTS -1025-rev | GAGGTAGTCAGTCAGGTCCC |
| C1-*β-Actin*-Δ1025 bp | *β-Actin*-HTS -16-few | atcgtccGGCTTCCTTTGTCCCCAATC |
|  | *β-Actin*-HTS -1025-rev | GAGGTAGTCAGTCAGGTCCC |
| C1-*β-Actin*-Δ1025 bp | *β-Actin*-HTS -17-few | atgcttcGGCTTCCTTTGTCCCCAATC |
|  | *β-Actin*-HTS -1025-rev | GAGGTAGTCAGTCAGGTCCC |
| C1-*β-Actin*-Δ1025 bp | *β-Actin*-HTS -18-few | aagcgtgGGCTTCCTTTGTCCCCAATC |
|  | *β-Actin*-HTS -1025-rev | GAGGTAGTCAGTCAGGTCCC |
| *FANCF*-OFF 1 | *FANCF*-OFF-1-few | acacagtTCTCAAGTCACCTGGATCGT |
|  | *FANCF*-OFF 1-rev | GCAGTGGCGTCTTAGTCG |
| *FANCF*-OFF 1 | *FANCF*-OFF-2-few | acagtcaTCTCAAGTCACCTGGATCGT |
|  | *FANCF*-OFF 1-rev | GCAGTGGCGTCTTAGTCG |
| *FANCF*-OFF 1 | *FANCF*-OFF-3-few | actctgaTCTCAAGTCACCTGGATCGT |
|  | *FANCF*-OFF 1-rev | GCAGTGGCGTCTTAGTCG |
| *FANCF*-OFF 2 | *FANCF*-OFF-4-few | actgactACACCCTCACTAAGCAGCTC |
|  | *FANCF*-OFF 2-rev | CACTCCCAAGAACCCTGGAA |
| *FANCF*-OFF 2 | *FANCF*-OFF-5-few | agactctACACCCTCACTAAGCAGCTC |
|  | *FANCF*-OFF 2-rev | CACTCCCAAGAACCCTGGAA |
| *FANCF*-OFF 2 | *FANCF*-OFF-6-few | agtcacaACACCCTCACTAAGCAGCTC |
|  | *FANCF*-OFF 2-rev | CACTCCCAAGAACCCTGGAA |
| *FANCF*-OFF 3 | *FANCF*-OFF-7-few | cagatcaTTCTGGAAGGACTCAGGCAG |
|  | *FANCF*-OFF 3-rev | GTAACGCGTTCACTCTGAGC |
| *FANCF*-OFF 3 | *FANCF*-OFF-8-few | cagacatTTCTGGAAGGACTCAGGCAG |
|  | *FANCF*-OFF 3-rev | GTAACGCGTTCACTCTGAGC |
| *FANCF*-OFF 3 | *FANCF*-OFF-9-few | cagagtcTTCTGGAAGGACTCAGGCAG |
|  | *FANCF*-OFF 3-rev | GTAACGCGTTCACTCTGAGC |
| *FANCF*-OFF 4 | *FANCF*-OFF-10-few | tcagagaGTAGGAGGTGGCAGGGTTTT |
|  | *FANCF*-OFF 4-rev | TACATGCATCACTGGAGCCA |
| *FANCF*-OFF 4 | *FANCF*-OFF-12-few | tgactgaGTAGGAGGTGGCAGGGTTTT |
|  | *FANCF*-OFF 4-rev | TACATGCATCACTGGAGCCA |
| *FANCF*-OFF 4 | *FANCF*-OFF-13-few | tgagactGTAGGAGGTGGCAGGGTTTT |
|  | *FANCF*-OFF 4-rev | TACATGCATCACTGGAGCCA |
| *FANCF*-OFF 5 | *FANCF*-OFF-14-few | catcagaAGAGTGGCATGCAACCTAGA |
|  | *FANCF*-OFF 5-rev | AGCAGGAGAAATGAGTCACTTT |
| *FANCF*-OFF 5 | *FANCF*-OFF-15-few | catgtctAGAGTGGCATGCAACCTAGA |
|  | *FANCF*-OFF 5-rev | AGCAGGAGAAATGAGTCACTTT |
| *FANCF*-OFF 5 | *FANCF*-OFF-16-few | cacaagtAGAGTGGCATGCAACCTAGA |
|  | *FANCF*-OFF 5-rev | AGCAGGAGAAATGAGTCACTTT |
| *FANCF*-OFF 6 | *FANCF*-OFF-17-few | cacactgTCAAATCCCGCCACCTACTT |
|  | *FANCF*-OFF 6-rev | CCTCTCACCAAACCCAGGAA |
| *FANCF*-OFF 6 | *FANCF*-OFF-18-few | cacttgaTCAAATCCCGCCACCTACTT |
|  | *FANCF*-OFF 6-rev | CCTCTCACCAAACCCAGGAA |
| *FANCF*-OFF 6 | *FANCF*-OFF-19-few | cactgatTCAAATCCCGCCACCTACTT |
|  | *FANCF*-OFF 6-rev | CCTCTCACCAAACCCAGGAA |
| *HEK2*-OFF 1 | *HEK2*-OFF-1-few | cagtactAGAACCTGGCTAAGCATTGC |
|  | *HEK2*-OFF 1-rev | TGGCACCAGACCCAAGTAAA |
| *HEK2*-OFF 1 | *HEK2*-OFF-2-few | cagtctaAGAACCTGGCTAAGCATTGC |
|  | *HEK2*-OFF 1-rev | TGGCACCAGACCCAAGTAAA |
| *HEK2*-OFF 1 | *HEK2*-OFF-3-few | ctagacaAGAACCTGGCTAAGCATTGC |
|  | *HEK2*-OFF 1-rev | TGGCACCAGACCCAAGTAAA |
| *HEK2*-OFF 2 | *HEK2*-OFF-4-few | ctctcagGGCCCAGGGTAAGACTCTAC |
|  | *HEK2*-OFF 2-rev | TAATAGCAGTGTGGTGGGCA |
| *HEK2*-OFF 2 | *HEK2*-OFF-5-few | ctctgtaGGCCCAGGGTAAGACTCTAC |
|  | *HEK2*-OFF 2-rev | TAATAGCAGTGTGGTGGGCA |
| *HEK2*-OFF 2 | *HEK2*-OFF-6-few | ctgatgtGGCCCAGGGTAAGACTCTAC |
|  | *HEK2*-OFF 2-rev | TAATAGCAGTGTGGTGGGCA |
| *HEK2*-OFF 3 | *HEK2*-OFF-7-few | ctgtagaCCTCTGCCCTGACCATGTAA |
|  | *HEK2*-OFF 3-rev | TCGGGTGATTCTGACGCATA |
| *HEK2*-OFF 3 | *HEK2*-OFF-8-few | gaacactCCTCTGCCCTGACCATGTAA |
|  | *HEK2*-OFF 3-rev | TCGGGTGATTCTGACGCATA |
| *HEK2*-OFF 3 | *HEK2*-OFF-9-few | gatctcaCCTCTGCCCTGACCATGTAA |
|  | *HEK2*-OFF 3-rev | TCGGGTGATTCTGACGCATA |
| *HEK2*-OFF 4 | *HEK2*-OFF-10-few | cttgagtCGTGAGCTCAACAGGTAAGA |
|  | *HEK2*-OFF 4-rev | CATCCAGGGAGAAGAGGCC |
| *HEK2*-OFF 4 | *HEK2*-OFF-11-few | ctcacgaCGTGAGCTCAACAGGTAAGA |
|  | *HEK2*-OFF 4-rev | CATCCAGGGAGAAGAGGCC |
| *HEK2*-OFF 4 | *HEK2*-OFF-12-few | ctcagacCGTGAGCTCAACAGGTAAGA |
|  | *HEK2*-OFF 4-rev | CATCCAGGGAGAAGAGGCC |
| *HEK2*-OFF 5 | *HEK2*-OFF-13-few | gactcacTTGAAGGAGAGGGGAACAGG |
|  | *HEK2*-OFF 5-rev | CAATGACACCTTTGGCCCAA |
| *HEK2*-OFF 5 | *HEK2*-OFF-14-few | gagtcgtTTGAAGGAGAGGGGAACAGG |
|  | *HEK2*-OFF 5-rev | CAATGACACCTTTGGCCCAA |
| *HEK2*-OFF 5 | *HEK2*-OFF-15-few | gtacagaTTGAAGGAGAGGGGAACAGG |
|  | *HEK2*-OFF 5-rev | CAATGACACCTTTGGCCCAA |
| *RNF2*-OFF 1 | *RNF2*-OFF-1-few | gtcgtgaAAGCCAGGCATAAAAGACCAC |
|  | *RNF2*-OFF 1-rev | CCTGCCAATGAAATCCAAAATGA |
| *RNF2*-OFF 1 | *RNF2*-OFF-2-few | gtgaacaAAGCCAGGCATAAAAGACCAC |
|  | *RNF2*-OFF 1-rev | CCTGCCAATGAAATCCAAAATGA |
| *RNF2*-OFF 1 | *RNF2*-OFF-3-few | gtgactcAAGCCAGGCATAAAAGACCAC |
|  | *RNF2*-OFF 1-rev | CCTGCCAATGAAATCCAAAATGA |
| *RNF2*-OFF 2 | *RNF2*-OFF-4-few | gtgtcaaTGGTTGTGTGTATGTGTGGTG |
|  | *RNF2*-OFF 2-rev | GTCTATCCCAGTCGCCCC |
| *RNF2*-OFF 2 | *RNF2*-OFF-5-few | aactgtcTGGTTGTGTGTATGTGTGGTG |
|  | *RNF2*-OFF 2-rev | GTCTATCCCAGTCGCCCC |
| *RNF2*-OFF 2 | *RNF2*-OFF-6-few | aacgtgtTGGTTGTGTGTATGTGTGGTG |
|  | *RNF2*-OFF 2-rev | GTCTATCCCAGTCGCCCC |
| *RNF2*-OFF 3 | *RNF2*-OFF-7-few | acaactgTGGTTACAAAATCATCAGCCTGT |
|  | *RNF2*-OFF 3-rev | GCAATAATGACCATGGGAGCT |
| *RNF2*-OFF 3 | *RNF2*-OFF-8-few | acatcgaTGGTTACAAAATCATCAGCCTGT |
|  | *RNF2*-OFF 3-rev | GCAATAATGACCATGGGAGCT |
| *RNF2*-OFF 3 | *RNF2*-OFF-9-few | acatgagTGGTTACAAAATCATCAGCCTGT |
|  | *RNF2*-OFF 3-rev | GCAATAATGACCATGGGAGCT |
| *RNF2*-OFF 4 | *RNF2*-OFF-10-few | aagagctGATCCGCTCCAATCTCCGT |
|  | *RNF2*-OFF 4-rev | GTGTTTTGACAGGGTTGCCT |
| *RNF2*-OFF 4 | *RNF2*-OFF-11-few | aagtcagGATCCGCTCCAATCTCCGT |
|  | *RNF2*-OFF 4-rev | GTGTTTTGACAGGGTTGCCT |
| *RNF2*-OFF 4 | *RNF2*-OFF-12-few | atcagtgGATCCGCTCCAATCTCCGT |
|  | *RNF2*-OFF 4-rev | GTGTTTTGACAGGGTTGCCT |
| *RNF2*-OFF 5 | *RNF2*-OFF-13-few | acacgtaAACACATCGCACCTGACATG |
|  | *RNF2*-OFF 5-rev | CCCTTCACAGCAGACAAACC |
| *RNF2*-OFF 5 | *RNF2*-OFF-14-few | acagatcAACACATCGCACCTGACATG |
|  | *RNF2*-OFF 5-rev | CCCTTCACAGCAGACAAACC |
| *RNF2*-OFF 5 | *RNF2*-OFF-15-few | acagcatAACACATCGCACCTGACATG |
|  | *RNF2*-OFF 5-rev | CCCTTCACAGCAGACAAACC |
| *RNF2*-OFF 6 | *RNF2*-OFF-16-few | actacgtAGAAGCAGCTTTCCCAATGG |
|  | *RNF2*-OFF 6-rev | AGCGCTGATCTACCTGACTT |
| *RNF2*-OFF 6 | *RNF2*-OFF-17-few | actagacAGAAGCAGCTTTCCCAATGG |
|  | *RNF2*-OFF 6-rev | AGCGCTGATCTACCTGACTT |
| *RNF2*-OFF 6 | *RNF2*-OFF-17-few | acttgcaAGAAGCAGCTTTCCCAATGG |
|  | *RNF2*-OFF 6-rev | AGCGCTGATCTACCTGACTT |

**Supplementary Note 1. Python script for HTS data analysis.**

file_path = '/Volumes/YSH/TR'

def get_files():

file_list = []

for filepath, dirnames, filenames in os.walk(file_path):

for filename in filenames:

file_list.append(os.path.join(filepath, filename))

print(os.path.join(filepath, filename))

return file_list

def counter(arr):

return Counter(arr)

def get_seq(fast_file_name):

"""get all sequences"""

with gzip.open(fast_file_name, 'rt') as fasta_file:

identifiers = []

lengths = []

# for seq_record in SeqIO.parse(fasta_file, 'fastq'):

for seq_record in SeqIO.parse(fasta_file, 'fastq'):

identifiers.append(seq_record.id)

lengths.append(str(seq_record.seq))

result_dic = counter(lengths)

new_result_dic = sorted(result_dic.items(), key=lambda d: d[1], reverse=True)

data = pd.DataFrame(new_result_dic, columns=['seq', 'count'])

writer = pd.ExcelWriter(fast_file_name.split('.')[0] +'_datas.xlsx')

data.to_excel(writer, index=False)

writer.save()

writer.close()

def get_tcc_seq(fast_file_name):

"""Get sequences starting with different categories"""

# print("current file name：{}".format(fast_file_name))

with gzip.open(fast_file_name, 'rt') as fasta_file:

lengths = []

cat_list = []

all_seqs = [str(fa.seq) for fa in SeqIO.parse(fasta_file, 'fastq')]

for seq_record in all_seqs:

if seq_record[:7] not in cat_list:

cat_list.append(seq_record[:7])

for cat in cat_list:

print("current file name：{}，sequence：{}".format(fast_file_name, cat))

exec('category_{} = []'.format(cat))

for seq_record in all_seqs:

if seq_record.startswith(cat):

exec('category_{}.append("{}")'.format(cat, seq_record))

code = """result_dic_{} = sorted(counter(category_{}).items(), key=lambda d: d[1], reverse=True)\nif result_dic_{}:\n seq1 = result_dic_{}[0][0]\n t_len = len(category_{})\n for i in result_dic_{}[:500]:\n seq2 = Seq(i[0])\n n_len = int(i[1])\n percent_match = (n_len / t_len) * 100\n alignments = pairwise2.align.localds(seq1, seq2, matrix, -10, -1)\n for alignment in alignments:\n mylog = open(fast_file_name.split(\'.\')[0] +\'_\'+ "{}" +\'_handle_datas.txt\', mode = \'a\',encoding=\'utf-8\')\n mylog.write(format_alignment(*alignment)+\'percent_match : \'+ str(n_len) + \'/' + str(t_len)+ \' = \' + str(percent_match)+"\\n")\n mylog.close()\n data = pd.DataFrame(result_dic_{}, columns=[\'seq\', \'count\'])\n writer = pd.ExcelWriter(fast_file_name.split(\'.\')[0] +\'_\'+ "{}" + \'_datas.xlsx\')\n data.to_excel(writer, index=False)\n writer.save()\n writer.close()"""

exec(code.format(cat, cat, cat, cat, cat, cat, cat, cat, cat))
